# Supplementary material for: Association between self-reported sleep duration and serum lipid profile in a middle-aged and elderly population in Taiwan: a community-based, cross-sectional study
Source: BMJ Open. 2017 Oct 30;7(10):e015964. doi: 10.1136/bmjopen-2017-015964 (PMC5665250; doi:10.1136/bmjopen-2017-015964)
Supplement: Author's manuscript [file bmjopen-2017-015964.draft_revisions.pdf]

BMJ Open

BMJ Open is committed to open peer review. As part of this commitment we make the peer review history of every article we publish publicly available.

When an article is published we post the peer reviewers' comments and the authors' responses online. We also post the versions of the paper that were used during peer review. These are the versions that the peer review comments apply to.

The versions of the paper that follow are the versions that were submitted during the peer review process. They are not the versions of record or the final published versions. They should not be cited or distributed as the published version of this manuscript.

BMJ Open is an open access journal and the full, final, typeset and author-corrected version of record of the manuscript is available on our site with no access controls, subscription charges or pay-per-view fees (http://bmjopen.bmj.com).

If you have any questions on BMJ Open's open peer review process please email editorial.bmjopen@bmj.com

BMJ Open

# Association between self-reported sleep duration and serum lipid profile in a middle-aged and elderly population in Taiwan: A community-based, cross-sectional study

| | |
|---|---|
| Journal: | *BMJ Open* |
| Manuscript ID | bmjopen-2017-015964 |
| Article Type: | Research |
| Date Submitted by the Author: | 13-Jan-2017 |
| Complete List of Authors: | Lin, Pu; Chang Gung Memorial Hospital Linkou Branch, Family Medicine<br>Chang, Kai-Ting; Chang Gung Memorial Hospital Linkou Branch, Family Medicine<br>Lin, Yan-An; Chang Gung Memorial Hospital Linkou Branch, Family Medicine<br>Chuang, Hai-Hua; Chang Gung Memorial Hospital Taipei Branch<br>Chen, Jau-Yuan; Chang Gung Memorial Hospital Linkou Branch, Family Medicine; Chang Gung University College of Medicine |
| <b>Primary Subject Heading</b>: | General practice / Family practice |
| Secondary Subject Heading: | Epidemiology, Health informatics |
| Keywords: | Cholesterol/Lipid, Family Health, Metabolism, Prevention, Sleep Disorders |
| | |

SCHOLARONE™
Manuscripts

For peer review only

- **Article Title**

Association between self-reported sleep duration and serum lipid profile in a middle-aged and elderly population in Taiwan: A community-based, cross-sectional study

- **Running title**

Association between sleep duration and serum lipid profile

- **Author names and affiliations**

Pu Lin[1], Kai-Ting Chang[1], Yan-An Lin[1], Hai-Hua Chuang[2, #], Jau-Yuan Chen[1, 3, *]

[1]Department of Family Medicine, Chang-Gung Memorial Hospital, Linkou Branch, Taiwan

[2]Department of Family Medicine, Chang-Gung Memorial Hospital, Taipei Branch, Taiwan

[3]College of Medicine, Chang Gung University, Taoyuan, Taiwan

[#] Same contribution as corresponding author

*Corresponding author

E-mail: welins@cgmh.org.tw (J.-Y. Chen)

Postal address : No. 5, Fuxing St., Guishan Dist., Taoyuan City 333, Taiwan (R.O.C.)

Phone: +886-975362672

Fax: +886-3-3287715

- **Format**

Word count: 2033

Number of black and white figures: 1

Number of Tables: 4

Number of References: 24



For peer review only

## ABSTRACT

**Objectives:** The association between sleep duration and serum lipid profile in the middle-aged and elderly is unclear. The aim of this study was to investigate and evaluate the relationships between sleep duration and levels of serum total cholesterol (TC), LDL cholesterol (LDL-C), HDL cholesterol (HDL-C), and triglycerides (TG) in these populations.

**Design:** Cross-sectional observational study.

**Setting:** Community-based investigation in Guishan township of northern Taiwan.

**Participants:** A total of 400 community-dwelling middle-aged and elderly individuals were enrolled. All participants underwent a baseline assessment in 2014, which included anthropometrics, blood samples, and self-administered questionnaires. Participants were divided into three groups based on their sleep duration.

**Outcome measures:** Multivariate logistic regression was used to obtain odds ratios (ORs) and 95% confidence intervals (CIs) to assess the relationship between sleep duration and lipid profiles.

**Results:** Participant mean age was 64.5 years old and 35.3% were men. Subjects with longer (>7 hours) and shorter (<6 hours) nightly sleep duration had a higher prevalence of low HDL-C levels (HDL <40 mg/dL) than those with moderate sleep duration (6-7 hours). Multivariate logistic regression revealed that, compared to individuals with sleep duration of 6-7 hours, the odds of having low HDL-C were 3.68 (95% CI = 1.59-8.49) greater for



For peer review only - http://bmjopen.bmj.com/site/about/guidelines.xhtml

individuals with sleep duration of <6 hours and 2.89 (95% CI = 1.10-7.61) greater for individuals with sleep duration of >7 hours.

**Conclusions:** There was a U-shaped relationship between sleep duration and HDL-C levels. Sleep duration over 7 hours or less than 6 hours increased the risk of low serum HDL-C levels.

**Keywords:** Cholesterol/Lipid, Family Health, Metabolism, Prevention, Sleep Disorders.

**Strengths and limitations of this study:**

■The results of the study show that sleep duration of 6-7 hours per night reduced the risk of abnormal serum lipid profiles.

■This is the first study to explore the associations between sleep duration and lipid profiles in middle-aged and elderly Taiwanese population.

■ We could not determine a causal relationship in our findings because this was a cross-sectional study.

■Lifestyle questionnaires were self-reported, which may limit the accuracy of measurements.

For peer review only

## INTRODUCTION

Several studies have identified that longer or shorter sleep duration may increase the mortality and morbidity risks from diabetes mellitus [1-3], obesity [4], hypertension [5] and coronary heart disease [6]. Some studies have shown a U-shaped association between sleep duration and major morbidities [3 7 8]. Dyslipidemia, such as high levels of total cholesterol (TC), low density lipoprotein cholesterol (LDL-C), and triglycerides (TG) and low levels of high density lipoprotein cholesterol (HDL-C), increases the risk of cardiovascular disease [9 10]. There is a lack of consensus on possible significant associations between sleep duration and serum lipid profiles, and there have been few studies on this issue in the Taiwanese population. Therefore, we investigated the relationship between sleep duration and levels of serum TC, LDL-C, HDL-C, and TG in a community-based study in Taiwan.

## MATERIALS AND METHODS

The present study was an observational, cross-sectional study. We enrolled 400 participants, including 141 men and 250 women, older than 50 years of age from the Guishan township of northern Taiwan in 2014. Data collection comprised two parts: a physical status examination and a self-administered questionnaire. For the physical status examination, height, weight, abdominal circumference, blood pressure, and blood samples were collected for all patients. Blood samples included the following items: lipid profiles (TC, LDL-C, HDL-C, and TG), aspartate aminotransferase (AST) levels, fasting plasma glucose (FPG)



For peer review only - http://bmjopen.bmj.com/site/about/guidelines.xhtml

levels, creatinine levels, and uric acid levels. The self-administered questionnaire included items related to smoking, drinking, exercise, sleep, and other lifestyle habits.

**Definitions and variables**

Based on the NCEP (National Cholesterol Education Program) ATP III guidelines, we set different cutoff points for each item of the serum lipid profiles (LDL-C: 130 mg/dL; TC: 200 mg/dL; HDL-C: 40 mg/dL; and TG: 150 mg/dL). Hyperlipidemia was defined as having LDL-C, TC, or TG levels above the cutoff point; or use of lipid lowering agents.

The questionnaire about sleep included the question: “What was your daily average sleep duration during the past month (not counting the times lying on the bed without sleeping)?”. We divided sleep duration into three groups: less than 6 hours (<6 hours), greater than or equal to 6 hours but less than or equal to 7 hours (6-7 hours), and more than 7 hours (>7 hours). Hypertension was defined as self-reported hypertension or the use of anti-hypertensive drugs. Diabetes was defined as self-reported diabetes mellitus or the use of oral anti-diabetic drugs or insulin. Alcohol drinking was defined as drinking alcohol greater than or equal to 2 days per week. Regular exercise was defined as jogging, doing gymnastics, mountain climbing, doing qigong, or other exercises on a habitual basis.

The institutional review board (IRB) approved the study and all patients provided written informed consent.

For peer review only



For peer review only - http://bmjopen.bmj.com/site/about/guidelines.xhtml

**Statistical analysis**

Adequate blood samples and complete questionnaires were collected from all participants. After data were collected, statistical analysis was conducted using the PASW 21.0 statistical package (SPSS Inc., Chicago, USA). A $p$-value <0.05 was considered statistically significant. Our statistical analysis included multiple steps. First, characteristics were compared across hyperlipidemia and sleep duration sub-groups using t-tests, one-way ANOVA, and chi-square tests. Homogeneity examination was performed with ANOVA, and heterogeneous variables were analyzed with the Brown-Forsythe test. Second, we measured correlations between serum lipid profiles and related risk factors. Third, logistic regression analyses were conducted to assess the relationship between sleep duration and risk of high LDL-C, high TC, low HDL-C, and high TG levels; additional regression models were adjusted for relevant covariates.

**RESULTS**

The characteristics of study subjects with and without hyperlipidemia are shown in Table 1. Among the included participants, 260 (65%) had hyperlipidemia. Body mass index, waist circumference, waist-to-height ratio, AST levels, fasting plasma glucose levels, and uric acid levels were significantly higher in the hyperlipidemia group.



For peer review only - http://bmjopen.bmj.com/site/about/guidelines.xhtml

**Table 1** Characteristics of study subjects with and without hyperlipidemia.

| Variables | No hyperlipidemia (n=140) | Hyperlipidemia (n=260) | p-value |
|---|---|---|---|
| Sex (male) | 57 (40.7) | 84 (32.3) | 0.101 |
| Age (years) | 65.4 ± 8.4 | 64.0 ± 8.4 | 0.072 |
| Current smoking | 9 (6.4) | 34 (13.1) | 0.043 |
| Alcohol drinking | 20 (14.3) | 55 (21.2) | 0.107 |
| Regular exercise | 116 (83.2) | 211 (81.2) | 0.681 |
| Hypertension | 64 (45.7) | 137 (52.7) | 0.209 |
| Diabetes | 28 (19.7) | 56 (21.5) | 0.238 |
| LDL-C (mg/dL) | 97.6 ± 17.7 | 129.6 ± 32.6 | <0.001 |
| TC (mg/dL) | 169.8 ± 21.1 | 211.9 ± 33.2 | <0.001 |
| HDL-C (mg/dL) | 55.4 ± 12.8 | 53.9 ± 14.5 | 0.161 |
| TG (mg/dL) | 84.5 ± 29.3 | 142.3 ± 71.6 | <0.001 |
| Sleep (hours) | 6.2 ± 1.2 | 6.2 ± 1.3 | 0.276 |
| SBP (mmHg) | 128.0 ± 16.0 | 130.2 ± 17.0 | 0.267 |
| DBP (mmHg) | 76.1 ± 11.3 | 77.1 ± 12.2 | 0.592 |
| BMI (kg/m$^2$) | 23.7 ± 3.4 | 25.0 ± 3.6 | 0.001 |
| WC (cm) | 82.9 ± 8.5 | 86.3 ± 10.1 | 0.001 |
| WHtR | 0.5 ± 0.1 | 0.5 ± 0.1 | 0.003 |
| ALT (mg/dL) | 20.3 ± 8.9 | 23.9 ± 14.6 | 0.032 |
| Creatinine (mg/dL) | 0.8 ± 0.6 | 0.8 ± 0.3 | 0.408 |
| FPG (mg/dL) | 92.2 ± 19.1 | 98.4 ± 28.5 | 0.028 |
| Uric acid (mg/dL) | 5.4 ± 1.3 | 5.9 ± 1.4 | 0.001 |

Data expressed as mean ± SD for continuous variables and n (%) for categorical variables.

Abbreviations: TC = total cholesterol; TG = triglyceride; LDL-C = low density lipoprotein; HDL-C = high density lipoprotein; Sleep = sleep duration; ALT = alanine transaminase; WC = waist circumference; WHtR = waist to height ratio; BMI = body mass index; FPG = fasting plasma glucose; SBP = systolic blood pressure; DBP = diastolic blood pressure.

For peer review only



For peer review only - http://bmjopen.bmj.com/site/about/guidelines.xhtml

The characteristics of study subjects categorized by sleep duration are shown in Table 2. There were 100 participants in the <6 hours group, 255 in the 6-7 hours group, and 45 in the >7 hours group. Low HDL-C levels significantly higher in the >7 hours group (31.1%) than other two groups (9.8% for the 6-7 hours group and 13% for the <6 hours group); post-hoc tests also showed the difference was significant.

For peer review only



For peer review only - http://bmjopen.bmj.com/site/about/guidelines.xhtml

**Table 2** Characteristics of study subjects by sleep duration groups.

| Variable | <6 hours (n = 100) | 6-7 hours (n = 255) | >7 hours (n = 45) | p-value |
|---|---|---|---|---|
| Sex (male) | 30 (30) | 88 (34.5) | 23 (51.1) | 0.044 |
| Age (years) | 65.2 ± 7.6 | 64.1 ± 8.3 | 65.1 ± 10.6 | 0.530 |
| High LDL-C | 33 (33) | 87 (34.1) | 15 (33.3) | 0.978 |
| High TC | 49 (49) | 125 (49.0) | 21 (47.7) | 0.941 |
| Low HDL-C | 13 (13) | 25 (9.8) | 14 (31.1) | 0.001 |
| High TG | 22 (22) | 58 (22.7) | 14 (31.1) | 0.437 |
| Alcohol drinking | 15 (15) | 52 (20.4) | 11 (24.4) | 0.346 |
| Current Smoking | 10 (10) | 27 (10.6) | 6 (13.3) | 0.828 |
| Regular exercise | 80 (80) | 209 (81.9) | 39 (86.4) | 0.642 |
| Hypertension | 47 (47) | 125 (49.0) | 29 (64.4) | 0.122 |
| Diabetes | 25 (25) | 45 (17.6) | 9 (20.0) | 0.293 |
| Dyslipidemia | 69 (69) | 161 (63.1) | 30 (66.7) | 0.564 |
| SBP (mmHg) | 129.9 ± 16.5 | 128.6 ± 16.7 | 133.3 ± 25.2 | 0.216 |
| DBP (mmHg) | 76.4 ± 13.0 | 76.6 ± 11.8 | 78.9 ± 9.13 | 0.436 |
| BMI (kg/m$^2$) | 24.3 ± 3.9 | 24.6 ± 3.4 | 25.1 ± 3.6 | 0.394 |
| WC (cm) | 84.7 ± 10.4 | 84.8 ± 9.6 | 87.4 ± 8.6 | 0.235 |
| WHtR | 0.5 ± 0.1 | 0.6 ± 0.1 | 0.6 ± 0.1 | 0.417 |
| ALT (mg/dL) | 22.5 ± 14.0 | 22.3 ± 11.94 | 22.7 ± 15.9 | 0.530 |
| Creatinine (mg/dL) | 0.8 ± 0.3 | 0.8 ± 0.3 | 1.0 ± 0.9 | 0.920 |
| FPG (mg/dL) | 98.2 ± 28.2 | 94.8 ± 19.3 | 100.3 ± 45.1 | 0.519 |
| Uric acid (mg/dL) | 5.6 ± 1.3 | 5.8 ± 1.4 | 6.1 ± 1.4 | 0.101 |

Data expressed as mean ± SD for continuous variables and n (%) for categorical variables. Abbreviations: High TC = total cholesterol ≥200 (mg/d); High TG = triglyceride ≥150 (mg/d); High LDL-C = low density lipoprotein ≥130 (mg/d); Low HDL-C = high density lipoprotein ≤40 (mg/d); ALT = alanine transaminase; WC = waist circumference; WHtR = waist to height ratio; BMI = body mass index; FPG = fasting plasma glucose; SBP = systolic blood pressure; DBP = diastolic blood pressure.

For peer review only



For peer review only - http://bmjopen.bmj.com/site/about/guidelines.xhtml

Figures 1 show comparisons of serum lipid profiles by sleep duration for two age groups, age equal to or greater than 65 years old (elderly group) and all others (middle age group). Abnormal total cholesterol levels had highest prevalence (around 50%) among the four items; there were no significant differences by sleep duration (Figure 1A). The average prevalence of high LDL-C level was about one-third, and prevalence gradually increased with longer sleep duration in middle age group but decreased with longer sleep duration in the elderly group (Figure 1B). There was a U-shaped (or J-shaped) distribution in the association of low HDL-C levels with sleep duration; both age groups showed significantly higher prevalence of low HDL-C if sleep duration was <6 hours or >7 hours (Figure 1D). Abnormal triglyceride levels by sleep duration in the middle age group also appeared U-shaped (Figure 1C).

Spearman's correlation test showed low correlation among the serum lipid profile variables, except HDL-C with sex and waist circumference (Table 3). However, HDL-C was the only variable of the serum lipid profile significantly correlated with sleep duration.

For peer review only



For peer review only - http://bmjopen.bmj.com/site/about/guidelines.xhtml

**Table 3** Spearman's correlation coefficients between serum lipid profiles and related risk factors.

| | TC | LDL | HDL | TG |
|---|---|---|---|---|
| Sex | 0.247* | 0.159* | 0.365* | -0.012 |
| Age (years) | -0.098* | -0.089 | -0.072 | -0.008 |
| Current smoking | 0.053 | 0.047 | -0.133* | 0.138* |
| Alcohol | 0.089 | 0.076 | 0.016 | 0.01 |
| Exercise | -0.052 | -0.037 | -0.089 | 0.065 |
| BMI (kg/m$^2$) | 0.007 | 0.38 | -0.263* | 0.254* |
| FPG (mg/dL) | -0.126 | -0.127* | -0.255* | 0.268* |
| WC (cm) | -0.04 | 0.005 | -0.362* | 0.284* |
| Sleep (hours) | -0.04 | 0.007 | -0.122* | -0.001 |

*P-value <0.05

Data are correlation coefficients.

Abbreviations: WC=waist circumference; BMI=body mass index; FPG=fasting plasma glucose; Sleep= sleep duration.

For peer review only

Multivariable logistic regression model results are shown in Table 4. Model 1 assessed the crude odds ratio of lipid profiles by sleep duration; Model 2 adjusted for age; Model 3 adjusted for age and waist circumference; and Model 4 adjusted for age, waist circumference, and other traditional factors that influence lipid levels, including sex, alcohol drinking, exercise, and smoking. The 6–7-hour sleep duration group was set as the reference category. The p-value of the odds ratio were all above 0.05 (95% confidence interval including 1) when the outcome was high TC levels, high LDL-C levels, or high TG levels; the odds ratios were all significant in Models 1 through 4 when the dependent variable was low HDL-C levels.

For peer review only

101 **Table 4** Odds ratios for the associate between lipid profiles and reported sleep duration.

| | Sleep <6 hours (n=100) | Sleep 6-7 hours (n=255) | Sleep >7 hours (n=45) |
|---|---|---|---|
| | | Total cholesterol | |
| Model 1 | 1.02 (0.54-1.92) | 1.00 | 1.10 (0.54-2.22) |
| Model 2 | 0.99 (0.52-1.88) | 1.00 | 1.10 (0.52-2.24) |
| Model 3 | 1.01 (0.53-1.93) | 1.00 | 1.13 (0.56-2.31) |
| Model 4 | 0.90 (0.45-1.80) | 1.00 | 0.95 (0.44-2.02) |
| | | LDL-C | |
| Model 1 | 1.04 (0.53-2.03) | 1.00 | 0.99 (0.47-2.08) |
| Model 2 | 1.02 (0.52-2.00) | 1.00 | 0.99 (0.47-2.10) |
| Model 3 | 1.05 (0.53-2.07) | 1.00 | 1.03 (0.49-2.19) |
| Model 4 | 1.001(0.50-2.01) | 1.00 | 0.94 (0.43-2.04) |
| | | HDL-C | |
| Model 1 | 3.81 (1.80-8.05)* | 1.00 | 3.02 (1.28-7.14)* |
| Model 2 | 3.75 (1.77-7.93)* | 1.00 | 3.02 (1.28-7.16)* |
| Model 3 | 3.62 (1.63-8.00)* | 1.00 | 2.93 (1.17-7.34)* |
| Model 4 | 3.68 (1.59-8.49)* | 1.00 | 2.89 (1.10-7.61)* |
| | | Triglyceride | |
| Model 1 | 0.65 (0.33-1.31) | 1.00 | 0.63 (0.28-1.37) |
| Model 2 | 0.65 (0.33-1.31) | 1.00 | 0.63 (0.27-1.38) |
| Model 3 | 0.72 (0.35-1.46) | 1.00 | 0.69 (0.31-1.35) |
| Model 4 | 0.64 (0.31-1.34) | 1.00 | 0.60 (0.26-1.39) |

102 Data are expressed as odds ratio (OR) and 95% confidence interval (CI))

103 *P-value <0.05

104 †Model 1: unadjusted; Model 2: multiple logistic regression adjusted for age; Model 3: 105 multiple logistic regression adjusted for age and waist circumference; Model 4: multiple 106 logistic regression adjusted for age, sex, waist circumference, alcohol drinking, exercise, and 107 smoking.

108 Abbreviations: TC=total cholesterol; TG=triglyceride; LDL-C=low density lipoprotein; 109 HDL-C= high density lipoprotein.

For peer review only

## DISCUSSION

In our study, traditional risk factors, such as high body mass index, high waist circumference, high fasting plasma glucose levels, and high uric acid levels, were significantly associated with abnormal lipid values. Elevated AST levels may be associated with non-alcoholic liver disease. We found a U-shaped association between sleep duration and low HDL-C levels, and logistic regression models showed that sleep duration was significantly associated with low HDL-C levels regardless of adjustment for potential confounders.

Several studies have reported associations between serum lipid profiles and sleep duration, but the results of these studies have been inconsistent [11-16]. Choi et al. [11] collected data from 4,222 Korean participants over the age of 60 and found a U-shaped association between low HDL-C levels and high triglyceride levels, which were similar to the results of our study. Both short and long sleep durations were related to an increased risk of metabolic syndrome and sleep duration of 7 hours demonstrated the lowest prevalence of metabolic syndrome in this study. Hall et al. [12] reported that sleep duration was independently associated with three components of metabolic syndrome: abdominal obesity, elevated serum glucose levels, and elevated triglyceride levels; the optimal sleep duration for preventing metabolic syndrome determined by this study was 7 to 8 hours per night. Bjorvatn et al. [13] demonstrated that cholesterol levels, triglyceride levels, and blood pressure were higher in subjects with short


For peer review only - http://bmjopen.bmj.com/site/about/guidelines.xhtml

129 sleep duration. Another study [14] revealed that HDL-C levels decreased with short and long
130 sleep duration among normotensive, but not hypertensive, women.

131 The logistic regression models of low HDL-C level adjusted for age, sex, waist
132 circumference, alcohol drinking, exercise, and smoking. To our knowledge, the prevalence of
133 dyslipidemia is proportional to age and different in men and women. The association between
134 waist circumference and dyslipidemia has been confirmed in some studies [17 18]. Lifestyle
135 factors have been shown to influence serum lipid or lipoprotein levels. For example, smoking
136 decreases HDL-C levels and increases TG levels, whereas alcohol consumption increases the
137 levels of both. Exercise increases HDL-C levels and decreases TG levels. In addition, alcohol
138 consumption is reported to decrease LDL-C levels [19 20]. Even after adjusting for these
139 potential confounding factors, our logistic regression models showed significant differences
140 in odds of low HDL-C levels between sleep duration groups.

141 The influence that shorter sleep duration has on body weight and dyslipidemia has become
142 clearer in recent years. Sleep restriction is associated with hormone imbalance; it reduces leptin
143 (an appetite suppressant) and elevates ghrelin levels (an appetite stimulant) [4 21], which may
144 contribute to increased body weight and lead to dyslipidemia. The biochemical mechanism for
145 the relationship between longer sleeper and dyslipidemia has not been clearly confirmed; some
146 studies showed prolonged sleep duration may be associated with glucose intolerance and
147 diabetes [1 22]. Physical performance, such as reduced energy consumption due to increased time



For peer review only - http://bmjopen.bmj.com/site/about/guidelines.xhtml

in bed, may affect obesity; one study explained this relationship by showing that longer sleep duration was related to less exercise [6].

Some studies have reported that the relative risk of mortality and morbidity was lowest when sleep duration was between 7-8 hours per night [3 6-8], but other research has reported that 6-7 hours is more optimal [15]. The cutoff point of our study was set at 6-7 hours; we found the volunteers in our study generally woke up early regardless of the amount of time they slept, and they often had spontaneous arousal from nocturnal sleep. This may be explained by physiological age-related changes in circadian modulation, homeostatic factors, cardiopulmonary function, and endocrine function [23 24]; or be due to underlying chronic diseases. In an attempt to recommend the optimal sleep duration for lowering morbidity based on epidemiological data, it is inferred that the optimal sleep duration will vary for different populations [15].

Limitations of this study should be mentioned. First, we could not determine a causal relationship in our findings because this was a cross-sectional study. A cohort study is required for determining more causal relationships, which we aim to complete in the future. Second, selection bias might exist because the volunteer participant selection was limited to one township, and participants were not selected randomly or with a stratified method from the population. This could limit the generalizability of these results. In addition, our sample size was relatively small so sampling bias should be considered. Third, we did not group the



For peer review only - http://bmjopen.bmj.com/site/about/guidelines.xhtml

167 population by underlying disease, such as diabetes or cerebrovascular disease, due to limited
168 sample size; the proper cutoff for lipid profiles may differ according to underlying diseases.
169 Furthermore, the HDL-C cutoff point differs by sex; in metabolic syndrome, it is set as 40
170 mg/dL for men and 50 mg/dL for women. The influence of sleep duration on lipid metabolism
171 may also differ between men and women; for example, a study by Kaneita et al. [15] of 1,666
172 men and 2,329 women aged 20 years or older from Japan showed that HDL-C levels had a
173 U-shaped association with sleep duration in women but not men. Finally, the present study
174 estimated sleep duration only by a self-reported questionnaire, a more systematically biased
175 estimate than measured sleep duration.

176 **CONCLUSIONS**

177 In our study of a Taiwanese population, sleep duration of 6-7 hours per night reduced the
178 risk of abnormal serum lipid profiles. Sleep duration over 7 hours or less than 6 hours may
179 increase the risk of low serum HDL-C levels, although adjustment for risk factors attenuated
180 this relationship. Inappropriate sleep duration might be a potential risk factor for low HDL-C
181 levels, and adequate sleep duration may improve low HDL-C status. Lifestyle interventions,
182 including exercise and abstaining from alcohol and smoking, should be initiated early in
183 high-risk groups.



For peer review only - http://bmjopen.bmj.com/site/about/guidelines.xhtml

For peer review only

**Contributors**

PL was involved in writing of the manuscript, collection and analyzed the data. KTC and YAL were involved in collection data. HHC and JYC contributed conceived, designed and performed the experiments, collected and analyzed the data, revising it critically for important intellectual content and final approval of the version to be submitted.

**Funding** This work was supported by Chang Gung Memorial Hospital (CORPG3C0011, CORPG3C0171, CORPG3C0172, CZRPG3C0053).

**Competing interests** None declared.

**Ethics approval** The study was approved by Chang-Gung Medical Foundation Institutional Review Board (102-2304B), and written informed consent was given by all the participants before enrollment.

**Data sharing statement** No additional data are available.

For peer review only



For peer review only - http://bmjopen.bmj.com/site/about/guidelines.xhtml

## REFERENCES

1. Gottlieb DJ, Punjabi NM, Newman AB, et al. Association of sleep time with diabetes mellitus and impaired glucose tolerance. *Archives of internal medicine* 2005;165(8):863-7. doi: 10.1001/archinte.165.8.863 [published Online First: 2005/04/27]

2. Yaggi HK, Araujo AB, McKinlay JB. Sleep duration as a risk factor for the development of type 2 diabetes. *Diabetes care* 2006;29(3):657-61. [published Online First: 2006/03/01]

3. Tamakoshi A, Ohno Y. Self-reported sleep duration as a predictor of all-cause mortality: results from the JACC study, Japan. *Sleep* 2004;27(1):51-4. [published Online First: 2004/03/05]

4. Taheri S, Lin L, Austin D, et al. Short sleep duration is associated with reduced leptin, elevated ghrelin, and increased body mass index. *PLoS medicine* 2004;1(3):e62. doi: 10.1371/journal.pmed.0010062 [published Online First: 2004/12/17]

5. Gottlieb DJ, Redline S, Nieto FJ, et al. Association of usual sleep duration with hypertension: the Sleep Heart Health Study. *Sleep* 2006;29(8):1009-14. [published Online First: 2006/09/02]

6. Ayas NT, White DP, Manson JE, et al. A prospective study of sleep duration and coronary heart disease in women. *Archives of internal medicine* 2003;163(2):205-9. [published Online First: 2003/01/28]

7. Kripke DF, Garfinkel L, Wingard DL, et al. Mortality associated with sleep duration and



For peer review only - http://bmjopen.bmj.com/site/about/guidelines.xhtml

insomnia. *Archives of general psychiatry* 2002;59(2):131-6. [published Online First: 2002/02/05]

8. Kripke DF, Simons RN, Garfinkel L, et al. Short and long sleep and sleeping pills. Is increased mortality associated? *Archives of general psychiatry* 1979;36(1):103-16. [published Online First: 1979/01/01]

9. Third Report of the National Cholesterol Education Program (NCEP) Expert Panel on Detection, Evaluation, and Treatment of High Blood Cholesterol in Adults (Adult Treatment Panel III) final report. *Circulation* 2002;106(25):3143-421. [published Online First: 2002/12/18]

10. Strand LB, Tsai MK, Gunnell D, et al. Self-reported sleep duration and coronary heart disease mortality: A large cohort study of 400,000 Taiwanese adults. *International journal of cardiology* 2016;207:246-51. doi: 10.1016/j.ijcard.2016.01.044 [published Online First: 2016/01/26]

11. Choi KM, Lee JS, Park HS, et al. Relationship between sleep duration and the metabolic syndrome: Korean National Health and Nutrition Survey 2001. *International journal of obesity (2005)* 2008;32(7):1091-7. doi: 10.1038/ijo.2008.62 [published Online First: 2008/05/14]

12. Hall MH, Muldoon MF, Jennings JR, et al. Self-reported sleep duration is associated with the metabolic syndrome in midlife adults. *Sleep* 2008;31(5):635-43. [published Online

For peer review only



For peer review only - http://bmjopen.bmj.com/site/about/guidelines.xhtml

First: 2008/06/04]

13. Bjorvatn B, Sagen IM, Oyane N, et al. The association between sleep duration, body mass index and metabolic measures in the Hordaland Health Study. *Journal of sleep research* 2007;16(1):66-76. doi: 10.1111/j.1365-2869.2007.00569.x [published Online First: 2007/02/21]

14. Williams CJ, Hu FB, Patel SR, et al. Sleep duration and snoring in relation to biomarkers of cardiovascular disease risk among women with type 2 diabetes. *Diabetes care* 2007;30(5):1233-40. doi: 10.2337/dc06-2107 [published Online First: 2007/02/27]

15. Kaneita Y, Uchiyama M, Yoshiike N, et al. Associations of usual sleep duration with serum lipid and lipoprotein levels. *Sleep* 2008;31(5):645-52. [published Online First: 2008/06/04]

16. van den Berg JF, Miedema HM, Tulen JH, et al. Long sleep duration is associated with serum cholesterol in the elderly: the Rotterdam Study. *Psychosomatic medicine* 2008;70(9):1005-11. doi: 10.1097/PSY.0b013e318186e656 [published Online First: 2008/10/10]

17. Manjareeka M, Nanda S, Mishra J, et al. Correlation between anthropometry and lipid profile in healthy subjects of Eastern India. *Journal of mid-life health* 2015;6(4):164-8. doi: 10.4103/0976-7800.172302 [published Online First: 2016/02/24]

18. Lee S, Bacha F, Arslanian SA. Waist circumference, blood pressure, and lipid components

For peer review only

of the metabolic syndrome. *The Journal of pediatrics* 2006;149(6):809-16. doi: 10.1016/j.jpeds.2006.08.075 [published Online First: 2006/12/02]

19. Hata Y, Nakajima K. Life-style and serum lipids and lipoproteins. *Journal of atherosclerosis and thrombosis* 2000;7(4):177-97. [published Online First: 2001/08/28]

20. Choudhury SR, Ueshima H, Kita Y, et al. Alcohol intake and serum lipids in a Japanese population. *International journal of epidemiology* 1994;23(5):940-7. [published Online First: 1994/10/01]

21. Spiegel K, Tasali E, Penev P, et al. Brief communication: Sleep curtailment in healthy young men is associated with decreased leptin levels, elevated ghrelin levels, and increased hunger and appetite. *Annals of internal medicine* 2004;141(11):846-50. [published Online First: 2004/12/08]

22. Ayas NT, White DP, Al-Delaimy WK, et al. A prospective study of self-reported sleep duration and incident diabetes in women. *Diabetes care* 2003;26(2):380-4. [published Online First: 2003/01/28]

23. Espiritu JR. Aging-related sleep changes. *Clinics in geriatric medicine* 2008;24(1):1-14, v. doi: 10.1016/j.cger.2007.08.007 [published Online First: 2007/11/24]

24. Edwards BA, O'Driscoll DM, Ali A, et al. Aging and sleep: physiology and pathophysiology. *Seminars in respiratory and critical care medicine* 2010;31(5):618-33. doi: 10.1055/s-0030-1265902 [published Online First: 2010/10/14]



For peer review only - http://bmjopen.bmj.com/site/about/guidelines.xhtml

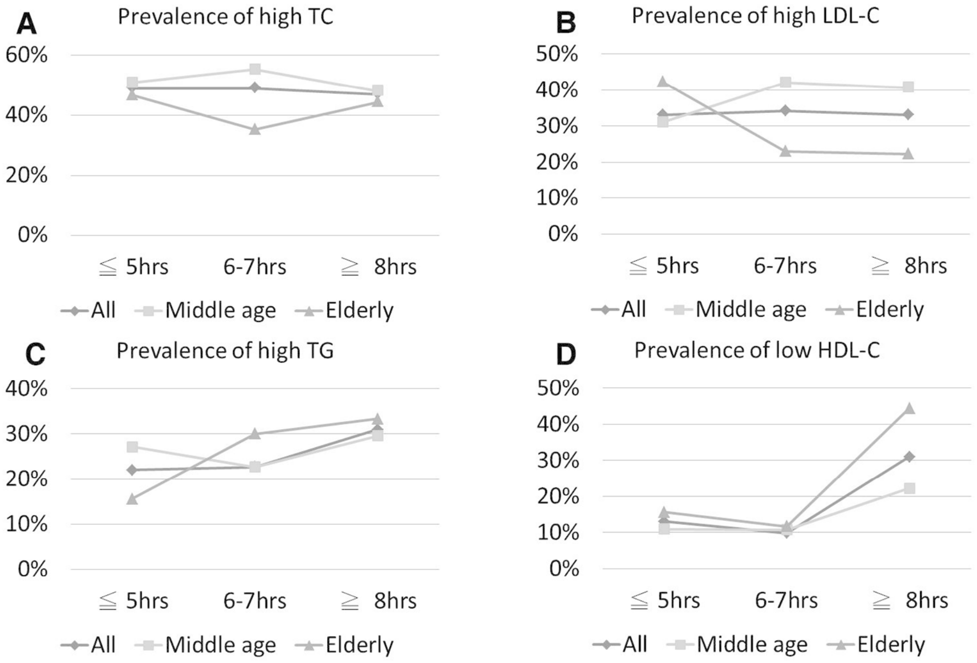


**Figure1** Prevalence of serum lipid profiles by sleep duration between two age groups (middle age: 50-65 years old; elderly: ≥65 years old)

Figure1 Prevalence of serum lipid profiles by sleep duration between two age groups (middle age: 50-65 years old; elderly: ≥65 years old)

256x200mm (150 x 150 DPI)

w only

**STROBE 2007 (v4) Statement—Checklist of items that should be included in reports of *cross-sectional studies***

| Section/Topic | Item # | Recommendation | Reported on page # |
|---|---|---|---|
| **Title and abstract** | 1 | (*a*) Indicate the study's design with a commonly used term in the title or the abstract | 1-2 |
| | | (*b*) Provide in the abstract an informative and balanced summary of what was done and what was found | 2-3 |
| **Introduction** | | | |
| Background/rationale | 2 | Explain the scientific background and rationale for the investigation being reported | 4 |
| Objectives | 3 | State specific objectives, including any prespecified hypotheses | 4 |
| **Methods** | | | |
| Study design | 4 | Present key elements of study design early in the paper | 4 |
| Setting | 5 | Describe the setting, locations, and relevant dates, including periods of recruitment, exposure, follow-up, and data collection | 4 |
| Participants | 6 | (*a*) Give the eligibility criteria, and the sources and methods of selection of participants | NA |
| Variables | 7 | Clearly define all outcomes, exposures, predictors, potential confounders, and effect modifiers. Give diagnostic criteria, if applicable | 5 |
| Data sources/ measurement | 8* | For each variable of interest, give sources of data and details of methods of assessment (measurement). Describe comparability of assessment methods if there is more than one group | 4-5 |
| Bias | 9 | Describe any efforts to address potential sources of bias | NA |
| Study size | 10 | Explain how the study size was arrived at | 4 |
| Quantitative variables | 11 | Explain how quantitative variables were handled in the analyses. If applicable, describe which groupings were chosen and why | 6 |
| Statistical methods | 12 | (*a*) Describe all statistical methods, including those used to control for confounding | 6 |
| | | (*b*) Describe any methods used to examine subgroups and interactions | NA |
| | | (*c*) Explain how missing data were addressed | NA |
| | | (*d*) If applicable, describe analytical methods taking account of sampling strategy | NA |

For peer review only - http://bmjopen.bmj.com/site/about/guidelines.xhtml

| | | | |
|---|---|---|---|
| | | (*e*) Describe any sensitivity analyses | NA |
| **Results** | | | |
| Participants | 13* | (a) Report numbers of individuals at each stage of study—eg numbers potentially eligible, examined for eligibility, confirmed eligible, included in the study, completing follow-up, and analysed | NA |
| | | (b) Give reasons for non-participation at each stage | NA |
| | | (c) Consider use of a flow diagram | NA |
| Descriptive data | 14* | (a) Give characteristics of study participants (eg demographic, clinical, social) and information on exposures and potential confounders | 7-9 |
| | | (b) Indicate number of participants with missing data for each variable of interest | NA |
| Outcome data | 15* | Report numbers of outcome events or summary measures | 10-12 |
| Main results | 16 | (*a*) Give unadjusted estimates and, if applicable, confounder-adjusted estimates and their precision (eg, 95% confidence interval). Make clear which confounders were adjusted for and why they were included | 12-13 |
| | | (*b*) Report category boundaries when continuous variables were categorized | NA |
| | | (*c*) If relevant, consider translating estimates of relative risk into absolute risk for a meaningful time period | NA |
| Other analyses | 17 | Report other analyses done—eg analyses of subgroups and interactions, and sensitivity analyses | NA |
| **Discussion** | | | |
| Key results | 18 | Summarise key results with reference to study objectives | 14 |
| Limitations | 19 | Discuss limitations of the study, taking into account sources of potential bias or imprecision. Discuss both direction and magnitude of any potential bias | 16-17 |
| Interpretation | 20 | Give a cautious overall interpretation of results considering objectives, limitations, multiplicity of analyses, results from similar studies, and other relevant evidence | 14-17 |
| Generalisability | 21 | Discuss the generalisability (external validity) of the study results | NA |
| **Other information** | | | |
| Funding | 22 | Give the source of funding and the role of the funders for the present study and, if applicable, for the original study on which the present article is based | 18 |

*Give information separately for cases and controls in case-control studies and, if applicable, for exposed and unexposed groups in cohort and cross-sectional studies.

**Note:** An Explanation and Elaboration article discusses each checklist item and gives methodological background and published examples of transparent reporting. The STROBE checklist is best used in conjunction with this article (freely available on the Web sites of PLoS Medicine at http://www.plosmedicine.org/, Annals of Internal Medicine at http://www.annals.org/, and Epidemiology at http://www.epidem.com/). Information on the STROBE Initiative is available at www.strobe-statement.org.

For peer review only

BMJ Open

# Association between self-reported sleep duration and serum lipid profile in a middle-aged and elderly population in Taiwan: A community-based, cross-sectional study

| | |
|---|---|
| Journal: | *BMJ Open* |
| Manuscript ID | bmjopen-2017-015964.R1 |
| Article Type: | Research |
| Date Submitted by the Author: | 16-Jun-2017 |
| Complete List of Authors: | Lin, Pu; Chang Gung Memorial Hospital Linkou Branch, Family Medicine<br>Chang, Kai-Ting; Chang Gung Memorial Hospital Linkou Branch, Family Medicine<br>Lin, Yan-An; Chang Gung Memorial Hospital Linkou Branch, Family Medicine<br>Tzeng, I-Shiang; Taipei Tzu Chi General Hospital, Research<br>Chuang, Hai-Hua; Chang Gung Memorial Hospital Taipei Branch<br>Chen, Jau-Yuan; Chang Gung Memorial Hospital Linkou Branch, Family Medicine; Chang Gung University College of Medicine |
| <b>Primary Subject Heading</b>: | General practice / Family practice |
| Secondary Subject Heading: | Epidemiology, Health informatics |
| Keywords: | Cholesterol/Lipid, Family Health, Metabolism, Prevention, Sleep Disorders |
| | |

SCHOLARONE™
Manuscripts

For peer review only

- **Article Title**

Association between self-reported sleep duration and serum lipid profile in a middle-aged and elderly population in Taiwan: A community-based, cross-sectional study

- **Running title**

Association between sleep duration and serum lipid profile

- **Author names and affiliations**

Pu Lin[1], Kai-Ting Chang[1], Yan-An Lin[1], I-Shiang Tzeng[2], Hai-Hua Chuang[3, #], Jau-Yuan Chen[1, 4, *]

[1]Department of Family Medicine, Chang-Gung Memorial Hospital, Linkou Branch, Taiwan

[2]Department of Research, Taipei Tzu Chi Hospital, Buddhist Tzu Chi Medical Foundation, New Taipei city, Taiwan

[3]Department of Family Medicine, Chang-Gung Memorial Hospital, Taipei Branch, Taiwan

[4]College of Medicine, Chang Gung University, Taoyuan, Taiwan

[#] Same contribution as corresponding author

*Corresponding author

E-mail: welins@cgmh.org.tw (J.-Y. Chen)

Postal address : No. 5, Fuxing St., Guishan Dist., Taoyuan City 333, Taiwan (R.O.C.)

Phone: +886-975362672

Fax: +886-3-3287715

- **Format**

Word count: 2369

Number of black and white figures: 1



For peer review only - http://bmjopen.bmj.com/site/about/guidelines.xhtml

For peer review only

Number of Tables: 3

Number of References: 36

**ABSTRACT**

**Objectives:** The association between sleep duration and serum lipid profile in the middle-aged and elderly is unclear. The aim of this study was to investigate and evaluate the relationships between sleep duration and levels of serum total cholesterol (TC), LDL cholesterol (LDL-C), HDL cholesterol (HDL-C), and triglycerides (TG) in these populations.

**Design:** Cross-sectional observational study.

**Setting:** Community-based investigation in Guishan township of northern Taiwan.

**Participants:** A total of 400 community-dwelling middle-aged and elderly individuals were enrolled. All participants underwent a baseline assessment in 2014, which included anthropometrics, blood samples, and self-administered questionnaires. Participants were classified into three groups based on their sleep duration.

**Outcome measures:** Multivariate logistic regression was used to obtain odds ratios (ORs) and 95% confidence intervals (CIs) to assess the relationship between sleep duration and lipid profiles.

**Results:** Participant mean age was 64.5 years old and 35.3% were men. Subjects with longer (>7 hours) and shorter (<6 hours) nightly sleep duration had a higher prevalence of low HDL-C levels (HDL <40 mg/dL) than those with moderate sleep duration (6-7 hours).



For peer review only - http://bmjopen.bmj.com/site/about/guidelines.xhtml

Multivariate logistic regression revealed that, compared to individuals with sleep duration of 6-7 hours, the odds of having low HDL-C were 3.68 (95% CI = 1.59-8.49) greater for individuals with sleep duration of <6 hours and 2.89 (95% CI = 1.10-7.61) greater for individuals with sleep duration of >7 hours.

**Conclusions:** There was a U-shaped relationship between sleep duration and HDL-C levels. Sleep duration over 7 hours or less than 6 hours increased the risk of low serum HDL-C levels.

**Keywords:** Cholesterol/Lipid, Family Health, Metabolism, Prevention, Sleep Disorders.

**Strengths and limitations of this study:**

- The results of the study show that sleep duration of 6-7 hours per night reduced the risk of abnormal serum lipid profiles.
- This is the first study to explore the associations between sleep duration and lipid profiles in middle-aged and elderly Taiwanese population.
- We could not determine a causal relationship in our findings because this was a cross-sectional study.
- Lifestyle questionnaires were self-reported, which may limit the accuracy of measurements.



For peer review only - http://bmjopen.bmj.com/site/about/guidelines.xhtml

## INTRODUCTION

As we known, the most aged adult suffers lipid profile disorder.[1] Moreover, the different levels in percentage of lipid disorders may have association between demography variables (i.e. as age and gender).[1] A study also found significant association between high prevalence of dyslipidemia and risk factors, such as increasing age, smoking status, hypertension, diabetes, and body mass index.[2] Several studies have identified that longer or shorter sleep duration may increase the mortality and morbidity risks from diabetes mellitus [3-5], obesity [6], hypertension [7] and coronary heart disease [8]. Some studies have shown a U-shaped association between sleep duration and major morbidities [5 9 10]. Cardiovascular disease has been a leading cause of death worldwide.[11] Dyslipidemia, such as high levels of total cholesterol (TC), low density lipoprotein cholesterol (LDL-C), and triglycerides (TG) and low levels of high density lipoprotein cholesterol (HDL-C), increases the risk of cardiovascular disease[12], and hence remains an important issue in the field of health promotion and disease prevention. Previous studies show age-associated alterations in the level, composition, and function of lipid profiles, including an inverted U-shaped quadratic trajectory for TC, LDL-C, and TG[13 14], and a decrease anti-oxidative ability.[15] Other than age, factors that have been suggested to be related to lipid profile levels include BMI, body composition, diet, and cardiorespiratory fitness.[13 14]

Though some study suggests that sleep deprivation may bring changes to plasma lipid [16],there is a lack of consensus on possible significant associations between sleep duration and



For peer review only - http://bmjopen.bmj.com/site/about/guidelines.xhtml

serum lipid profiles, and even fewer data available in the Taiwanese population[17]. Therefore, we investigated the relationship between sleep duration and levels of serum TC, LDL-C, HDL-C, and TG in a community-based study in Taiwan.

## MATERIALS AND METHODS

### Study participants

The present study was an observational, cross-sectional study. We enrolled 400 participants, including 141 men and 259 women. The inclusion criteria included (1) the residents aged over 50 years old; (2) the residents living in Guishan township. 12 people aged younger than 50 years old were excluded from the study. Subjects were excluded if (1) the residents could not complete the full examinations or missing data for age, sex, anthropometric values and blood test results; (2) the residents were functionally dependent; (3) the residents were unable to adequately communicate with the interviewers; (4) the residents had major illness recently; (5) the residents with known sleep disorders.

### Data collection

Data collection comprised two parts: a physical status examination and a self-administered questionnaire. For the physical status examination, height, weight, abdominal circumference, blood pressure, and blood samples were collected for all participants. Blood samples included the following items: lipid profiles (TC, LDL-C, HDL-C,



For peer review only - http://bmjopen.bmj.com/site/about/guidelines.xhtml

For peer review only

and TG), aspartate aminotransferase (AST) levels, fasting plasma glucose (FPG) levels, creatinine levels, and uric acid levels. The self-administered questionnaire included items related to smoking, drinking, exercise, sleep, and other lifestyle habits. All participants were performed in the same day recorded from March 2014 to August 2014.

**Definitions and variables**

Based on the NCEP (National Cholesterol Education Program) ATP III guidelines, we set different cutoff points for each item of the serum lipid profiles (LDL-C: 130 mg/dL; TC: 200 mg/dL; HDL-C: 40 mg/dL; and TG: 150 mg/dL). Hyperlipidemia was defined as having LDL-C, TC, or TG levels above the cutoff point.

Applied from Pittsburgh Sleep Quality Index(PSQI)[17], the questionnaire about sleep included the question: "What was your daily average sleep duration during the past month (not counting the times lying on the bed without sleeping)?". We divided sleep duration into three groups: less than 6 hours (<6 hours), greater than or equal to 6 hours but less than or equal to 7 hours (6-7 hours), and more than 7 hours (>7 hours). Hypertension was defined as self-reported hypertension or the use of anti-hypertensive drugs. Diabetes was defined as self-reported diabetes mellitus or the use of oral anti-diabetic drugs or insulin. Alcohol drinking was defined as drinking alcohol greater than or equal to 2 days per week. Regular exercise was defined as exercise times were greater or equal to 2 days per week. Current smoking was defined as literal meaning.



For peer review only - http://bmjopen.bmj.com/site/about/guidelines.xhtml

The institutional review board (IRB) approved the study and all patients provided written informed consent.

**Statistical analysis**

Adequate blood samples and complete questionnaires were collected from all participants. After data were collected, statistical analysis was conducted using the SPSS 21.0 statistical package (SPSS Inc., Chicago, USA). A $p$-value <0.05 was considered statistically significant. Our statistical analysis included multiple steps. First, characteristics were compared across hyperlipidemia and sleep duration sub-groups using t-tests, one-way ANOVA, and chi-square tests. Homogeneity examination was performed with ANOVA, and heterogeneous variables were analyzed with the Brown-Forsythe test. Second, we measured correlations between serum lipid profiles and related risk factors. Third, logistic regression analyses were conducted to assess the relationship between sleep duration and risk of high LDL-C, high TC, low HDL-C, and high TG levels; additional regression models were adjusted for relevant covariates.

**RESULTS**

The characteristics of study subjects with and without hyperlipidemia are shown in Table 1. Among the included participants, 260 (65%) had hyperlipidemia. Body mass index, waist circumference, waist-to-height ratio, AST levels, fasting plasma glucose levels, and uric acid levels were significantly higher in the hyperlipidemia group.



For peer review only - http://bmjopen.bmj.com/site/about/guidelines.xhtml

**Table 1** Characteristics of study subjects with and without hyperlipidemia.

| Variables | No hyperlipidemia (n=140) | Hyperlipidemia (n=260) | p-value |
|---|---|---|---|
| Sex (male) | 57 (40.7) | 84 (32.3) | 0.101 |
| Age (years) | 65.4 ± 8.4 | 64.0 ± 8.4 | 0.072 |
| Current smoking | 9 (6.4) | 34 (13.1) | 0.043 |
| Alcohol drinking | 20 (14.3) | 55 (21.2) | 0.107 |
| Regular exercise | 116 (83.2) | 211 (81.2) | 0.681 |
| Hypertension | 64 (45.7) | 137 (52.7) | 0.209 |
| Diabetes | 28 (19.7) | 56 (21.5) | 0.238 |
| LDL-C (mg/dL) | 97.6 ± 17.7 | 129.6 ± 32.6 | <0.001 |
| TC (mg/dL) | 169.8 ± 21.1 | 211.9 ± 33.2 | <0.001 |
| HDL-C (mg/dL) | 55.4 ± 12.8 | 53.9 ± 14.5 | 0.161 |
| TG (mg/dL) | 84.5 ± 29.3 | 142.3 ± 71.6 | <0.001 |
| Sleep (hours) | 6.2 ± 1.2 | 6.2 ± 1.3 | 0.276 |
| SBP (mmHg) | 128.0 ± 16.0 | 130.2 ± 17.0 | 0.267 |
| DBP (mmHg) | 76.1 ± 11.3 | 77.1 ± 12.2 | 0.592 |
| BMI (kg/m$^2$) | 23.7 ± 3.4 | 25.0 ± 3.6 | 0.001 |
| WC (cm) | 82.9 ± 8.5 | 86.3 ± 10.1 | 0.001 |
| WHtR | 0.52 ± 0.05 | 0.54 ± 0.06 | 0.003 |
| ALT (mg/dL) | 20.3 ± 8.9 | 23.9 ± 14.6 | 0.032 |
| Creatinine (mg/dL) | 0.8 ± 0.6 | 0.8 ± 0.3 | 0.408 |
| FPG (mg/dL) | 92.2 ± 19.1 | 98.4 ± 28.5 | 0.028 |
| Uric acid (mg/dL) | 5.4 ± 1.3 | 5.9 ± 1.4 | 0.001 |

Data expressed as mean ± SD for continuous variables and n (%) for categorical variables.

Abbreviations: TC = total cholesterol; TG = triglyceride; LDL-C = low density lipoprotein; HDL-C = high density lipoprotein; Sleep = sleep duration; ALT = alanine transaminase; WC = waist circumference; WHtR = waist to height ratio; BMI = body mass index; FPG = fasting plasma glucose; SBP = systolic blood pressure; DBP = diastolic blood pressure.

For peer review only



For peer review only - http://bmjopen.bmj.com/site/about/guidelines.xhtml

The characteristics of study subjects categorized by sleep duration are shown in Table 2. There were 100 participants in the <6 hours group, 255 in the 6-7 hours group, and 45 in the >7 hours group. Low HDL-C levels significantly higher in the >7 hours group (31.1%) than other two groups (9.8% for the 6-7 hours group and 13% for the <6 hours group); post-hoc tests also showed the difference was significant (significant difference between <6 hours group and >7 hours group(p value = 0.009)、6-7 hours and >7 hours(p value=0.001).

For peer review only



For peer review only - http://bmjopen.bmj.com/site/about/guidelines.xhtml

88 **Table 2** Characteristics of study subjects by sleep duration groups.

| Variable | <6 hours (n = 100) | 6-7 hours (n = 255) | >7 hours (n = 45) | p-value |
|---|---|---|---|---|
| Sex (male) | 30 (30) | 88 (34.5) | 23 (51.1)*† | 0.044 |
| Age (years) | 65.2 ± 7.6 | 64.1 ± 8.3 | 65.1 ± 10.6 | 0.530 |
| LDL-C (mg/dL) | 115.8 ± 30.4 | 119.2 ± 31.3 | 119.6 ± 40.0 | 0.643 |
| TC (mg/dL) | 197.2 ± 34.9 | 197.0 ± 34.2 | 198.0 ± 45.4 | 0.984 |
| HDL-C (mg/dL) | 57.4 ± 15.8 | 53.9 ± 12.5 | 51.2 ± 16.4 | 0.025 |
| TG (mg/dL) | 120.4 ± 61.8 | 119.8 ± 62.7 | 138.5 ± 88.6 | 0.207 |
| High LDL-C | 33 (33) | 87 (34.1) | 15 (33.3) | 0.978 |
| High TC | 49 (49) | 125 (49.0) | 21 (47.7) | 0.941 |
| Low HDL-C | 13 (13) | 25 (9.8) | 14 (31.1)*† | 0.001 |
| High TG | 22 (22) | 58 (22.7) | 14 (31.1) | 0.437 |
| Alcohol drinking | 15 (15) | 52 (20.4) | 11 (24.4) | 0.346 |
| Current Smoking | 10 (10) | 27 (10.6) | 6 (13.3) | 0.828 |
| Regular exercise | 80 (80) | 209 (81.9) | 39 (86.4) | 0.642 |
| Hypertension | 47 (47) | 125 (49.0) | 29 (64.4) | 0.122 |
| Diabetes | 25 (25) | 45 (17.6) | 9 (20.0) | 0.293 |
| Hyperlipidemia | 69 (69) | 161 (63.1) | 30 (66.7) | 0.564 |
| SBP (mmHg) | 129.9 ± 16.5 | 128.6 ± 16.7 | 133.3 ± 25.2 | 0.216 |
| DBP (mmHg) | 76.4 ± 13.0 | 76.6 ± 11.8 | 78.9 ± 9.13 | 0.436 |
| BMI (kg/m$^2$) | 24.3 ± 3.9 | 24.6 ± 3.4 | 25.1 ± 3.6 | 0.394 |
| WC (cm) | 84.7 ± 10.4 | 84.8 ± 9.6 | 87.4 ± 8.6 | 0.235 |
| WHtR | 0.5 ± 0.1 | 0.6 ± 0.1 | 0.6 ± 0.1 | 0.417 |
| ALT (mg/dL) | 22.5 ± 14.0 | 22.3 ± 11.94 | 22.7 ± 15.9 | 0.530 |
| Creatinine (mg/dL) | 0.8 ± 0.3 | 0.8 ± 0.3 | 1.0 ± 0.9 | 0.920 |
| FPG (mg/dL) | 98.2 ± 28.2 | 94.8 ± 19.3 | 100.3 ± 45.1 | 0.519 |
| Uric acid (mg/dL) | 5.6 ± 1.3 | 5.8 ± 1.4 | 6.1 ± 1.4 | 0.101 |

89 Data expressed as mean ± SD for continuous variables and n (%) for categorical variables. *
90 p-value < 0.05 compared with <6 hours group, † p-value < 0.05 compared with 6-7 hours
91 group.
92 Abbreviations: High TC = total cholesterol ≥200 (mg/d); High TG = triglyceride ≥150 (mg/d);
93 High LDL-C = low density lipoprotein ≥130 (mg/d); Low HDL-C = high density
94 lipoprotein ≤40 (mg/d); ALT = alanine transaminase; WC = waist circumference; WHtR =
95 waist to height ratio; BMI = body mass index; FPG = fasting plasma glucose; SBP = systolic



For peer review only - http://bmjopen.bmj.com/site/about/guidelines.xhtml

blood pressure; DBP = diastolic blood pressure.

For peer review only



For peer review only - http://bmjopen.bmj.com/site/about/guidelines.xhtml

Figures 1 show comparisons of serum lipid profiles by sleep duration for two age groups, age equal to or greater than 65 years old (elderly group, 168 people) and all others (middle age group, 232 people). Abnormal total cholesterol levels had highest prevalence (around 50%) among the four items; there were no significant differences by sleep duration (Figure 1A). The average prevalence of high LDL-C level was about one-third, and prevalence gradually increased with longer sleep duration in middle age group but decreased with longer sleep duration in the elderly group (Figure 1B). There was a U-shaped (or J-shaped) distribution in the association of low HDL-C levels with sleep duration; both age groups showed significantly higher prevalence of low HDL-C if sleep duration was <6 hours or >7 hours (Figure 1C). Abnormal triglyceride levels by sleep duration in the middle age group also appeared U-shaped (Figure 1D).

For peer review only

Multivariable logistic regression model results are shown in Table 3. Model 1 assessed the crude odds ratio of lipid profiles by sleep duration; Model 2 adjusted for age; Model 3 adjusted for age and waist circumference; and Model 4 adjusted for age, waist circumference, and other traditional factors that influence lipid levels, including sex, alcohol drinking, exercise, and smoking. The 6–7-hour sleep duration group was set as the reference category. The p-value of the odds ratio were all above 0.05 (95% confidence interval including 1) when the outcome was high TC levels, high LDL-C levels, or high TG levels; the odds ratios were all significant in Models 1 through 4 when the dependent variable was low HDL-C levels.

For peer review only



For peer review only - http://bmjopen.bmj.com/site/about/guidelines.xhtml

**Table 3** Odds ratios for the associate between lipid profiles and reported sleep duration.

| | **Sleep <6 hours (n=100)** | **Sleep 6-7 hours (n=255)** | **Sleep >7 hours (n=45)** |
|---|---|---|---|
| Total cholesterol | | | |
| Model 1 | 1.02 (0.54-1.92) | 1.00 | 1.10 (0.54-2.22) |
| Model 2 | 0.99 (0.52-1.88) | 1.00 | 1.10 (0.52-2.24) |
| Model 3 | 1.01 (0.53-1.93) | 1.00 | 1.13 (0.56-2.31) |
| Model 4 | 0.90 (0.45-1.80) | 1.00 | 0.95 (0.44-2.02) |
| LDL-C | | | |
| Model 1 | 1.04 (0.53-2.03) | 1.00 | 0.99 (0.47-2.08) |
| Model 2 | 1.02 (0.52-2.00) | 1.00 | 0.99 (0.47-2.10) |
| Model 3 | 1.05 (0.53-2.07) | 1.00 | 1.03 (0.49-2.19) |
| Model 4 | 1.001(0.50-2.01) | 1.00 | 0.94 (0.43-2.04) |
| HDL-C | | | |
| Model 1 | 3.81 (1.80-8.05)* | 1.00 | 3.02 (1.28-7.14)* |
| Model 2 | 3.75 (1.77-7.93)* | 1.00 | 3.02 (1.28-7.16)* |
| Model 3 | 3.62 (1.63-8.00)* | 1.00 | 2.93 (1.17-7.34)* |
| Model 4 | 3.68 (1.59-8.49)* | 1.00 | 2.89 (1.10-7.61)* |
| Triglyceride | | | |
| Model 1 | 0.65 (0.33-1.31) | 1.00 | 0.63 (0.28-1.37) |
| Model 2 | 0.65 (0.33-1.31) | 1.00 | 0.63 (0.27-1.38) |
| Model 3 | 0.72 (0.35-1.46) | 1.00 | 0.69 (0.31-1.35) |
| Model 4 | 0.64 (0.31-1.34) | 1.00 | 0.60 (0.26-1.39) |

Data are expressed as odds ratio (OR) and 95% confidence interval (CI))

*P-value <0.05

†Model 1: unadjusted; Model 2: multiple logistic regression adjusted for age; Model 3: multiple logistic regression adjusted for age and waist circumference; Model 4: multiple logistic regression adjusted for age, sex, waist circumference, alcohol drinking, exercise, and smoking.

Abbreviations: TC=total cholesterol; TG=triglyceride; LDL-C=low density lipoprotein; HDL-C= high density lipoprotein.

For peer review only



For peer review only - http://bmjopen.bmj.com/site/about/guidelines.xhtml

## DISCUSSION

In our study, traditional risk factors, such as high body mass index, high waist circumference, high fasting plasma glucose levels, and high uric acid levels, were significantly associated with abnormal lipid values. Elevated AST levels may be associated with non-alcoholic liver disease. We found a U-shaped association between sleep duration and low HDL-C levels, and logistic regression models showed that sleep duration was significantly associated with low HDL-C levels regardless of adjustment for potential confounders.

Several studies have reported associations between serum lipid profiles and sleep duration, but the results of these studies have been inconsistent [18-23]. Choi et al. [18] collected data from 4,222 Korean participants over the age of 60 and found a U-shaped association between low HDL-C levels and high triglyceride levels, which were similar to the results of our study. Both short and long sleep durations were related to an increased risk of metabolic syndrome and sleep duration of 7 hours demonstrated the lowest prevalence of metabolic syndrome in this study. Hall et al. [19] reported that sleep duration was independently associated with three components of metabolic syndrome: abdominal obesity, elevated serum glucose levels, and elevated triglyceride levels; the optimal sleep duration for preventing metabolic syndrome determined by this study was 7 to 8 hours per night. Bjorvatn et al. [20] demonstrated that cholesterol levels, triglyceride levels, and blood pressure were higher in subjects with short



For peer review only - http://bmjopen.bmj.com/site/about/guidelines.xhtml

sleep duration. Another study [21] revealed that HDL-C levels decreased with short and long sleep duration among normotensive, but not hypertensive, women.

The logistic regression models of low HDL-C level adjusted for age, sex, waist circumference, alcohol drinking, exercise, and smoking. To our knowledge, the prevalence of dyslipidemia is proportional to age and different in men and women. The association between waist circumference and dyslipidemia has been confirmed in some studies [24 25]. Lifestyle factors have been shown to influence serum lipid or lipoprotein levels. For example, smoking decreases HDL-C levels and increases TG levels, whereas alcohol consumption increases the levels of both[26-28]. Exercise increases HDL-C levels and decreases TG levels[29 30]. In addition, alcohol consumption is reported to decrease LDL-C levels [31 32]. Even after adjusting for these potential confounding factors, our logistic regression models showed significant differences in odds of low HDL-C levels between sleep duration groups.

The influence that shorter sleep duration has on body weight and dyslipidemia has become clearer in recent years. Sleep restriction is associated with hormone imbalance; it reduces leptin (an appetite suppressant) and elevates ghrelin levels (an appetite stimulant) [6 33], which may contribute to increased body weight and lead to dyslipidemia. The biochemical mechanism for the relationship between longer sleeper and dyslipidemia has not been clearly confirmed; some studies showed prolonged sleep duration may be associated with glucose intolerance and diabetes [3 34]. Physical performance, such as reduced energy consumption due to increased time


For peer review only - http://bmjopen.bmj.com/site/about/guidelines.xhtml

in bed, may affect obesity; one study explained this relationship by showing that longer sleep duration was related to less exercise [8].

Some studies have reported that the relative risk of mortality and morbidity was lowest when sleep duration was between 7-8 hours per night [5 8-10], but other research has reported that 6-7 hours is more optimal [22]. The cutoff point of our study was set at 6-7 hours; we found the volunteers in our study generally woke up early regardless of the amount of time they slept, and they often had spontaneous arousal from nocturnal sleep. This may be explained by physiological age-related changes in circadian modulation, homeostatic factors, cardiopulmonary function, and endocrine function [35 36]; or be due to underlying chronic diseases. In an attempt to recommend the optimal sleep duration for lowering morbidity based on epidemiological data, it is inferred that the optimal sleep duration will vary for different populations [22].

Limitations of this study should be mentioned. First, we could not determine a causal relationship in our findings because this was a cross-sectional study. A cohort study is required for determining more causal relationships, which we aim to complete in the future. Second, selection bias might exist because the volunteer participant selection was limited to one township, and participants were not selected randomly or with a stratified method from the population. This could limit the generalizability of these results. In addition, our sample size was relatively small so sampling bias should be considered. Third, we did not group the



For peer review only - http://bmjopen.bmj.com/site/about/guidelines.xhtml

182 population by underlying disease, such as diabetes or cerebrovascular disease, due to limited
183 sample size; the proper cutoff for lipid profiles may differ according to underlying diseases.
184 Furthermore, the HDL-C cutoff point differs by sex; in metabolic syndrome, it is set as 40
185 mg/dL for men and 50 mg/dL for women. The influence of sleep duration on lipid metabolism
186 may also differ between men and women; for example, a study by Kaneita et al. [22] of 1,666
187 men and 2,329 women aged 20 years or older from Japan showed that HDL-C levels had a
188 U-shaped association with sleep duration in women but not men. Fourth, glycosylated
189 hemoglobin might be a more appropriate measurement of diabetes instead of self-reported
190 diabetes mellitus or the use of oral anti-diabetic drugs or insulin. Fifth, as a majority of other
191 similar studies in the literature covering examining effects of sleep duration on serum lipids,
192 diet is not factored for as a covariate. It may be explained by daily life diet is a diversified
193 condition so that diet is difficult merging into one variable and bringing to the regression model
194 for adjustment. Sixth, lipid-lowering therapy was an important factor influencing lipid levels,
195 but we did not considering about lipid-lowering medication while formulating the
196 questionnaire. Finally, the present study estimated sleep duration only by a self-reported
197 questionnaire, a more systematically biased estimate than measured sleep duration.

198 **CONCLUSIONS**

199 In our study of a Taiwanese population, sleep duration of 6-7 hours per night reduced the
200 risk of abnormal serum lipid profiles. Sleep duration over 7 hours or less than 6 hours may



For peer review only - http://bmjopen.bmj.com/site/about/guidelines.xhtml

201 increase the risk of low serum HDL-C levels, although adjustment for risk factors attenuated this relationship. Inappropriate sleep duration might be a potential risk factor for low HDL-C levels, and adequate sleep duration may improve low HDL-C status. Lifestyle interventions, including exercise and abstaining from alcohol and smoking, should be initiated early in high-risk groups.

For peer review only



For peer review only - http://bmjopen.bmj.com/site/about/guidelines.xhtml

**Contributors**

PL was involved in writing of the manuscript, collection and analyzed the data. KTC and YAL were involved in collection data. IST provided statistical advice. HHC and JYC contributed conceived, designed and performed the experiments, collected and analyzed the data, revising it critically for important intellectual content and final approval of the version to be submitted.

**Funding** This work was supported by Chang Gung Memorial Hospital (CORPG3C0011, CORPG3C0171, CORPG3C0172, CZRPG3C0053).

**Competing interests** None declared.

**Ethics approval** The study was approved by Chang-Gung Medical Foundation Institutional Review Board (102-2304B), and written informed consent was given by all the participants before enrollment.

**Data sharing statement** No additional data are available.

For peer review only

## REFERENCES

1. Hoffmann K, Matyjaszczyk P, Zaporowska-Stachowiak I, et al. LIPID PROFILE OF POLISH PRIMARY CARE PATIENTS AGED 35-55 YEARS LIVING IN THE DISTRICT OF PLESZEW. *Acta poloniae pharmaceutica* 2016;73(2):537-46.

2. Ni W-Q, Liu X-L, Zhuo Z-P, et al. Serum lipids and associated factors of dyslipidemia in the adult population in Shenzhen. *Lipids in Health and Disease* 2015;14:71. doi: 10.1186/s12944-015-0073-7

3. Gottlieb DJ, Punjabi NM, Newman AB, et al. Association of sleep time with diabetes mellitus and impaired glucose tolerance. *Arch Intern Med* 2005;165(8):863-7. doi: 10.1001/archinte.165.8.863 [published Online First: 2005/04/27]

4. Yaggi HK, Araujo AB, McKinlay JB. Sleep duration as a risk factor for the development of type 2 diabetes. *Diabetes Care* 2006;29(3):657-61. [published Online First: 2006/03/01]

5. Tamakoshi A, Ohno Y. Self-reported sleep duration as a predictor of all-cause mortality: results from the JACC study, Japan. *Sleep* 2004;27(1):51-4. [published Online First: 2004/03/05]

6. Taheri S, Lin L, Austin D, et al. Short sleep duration is associated with reduced leptin, elevated ghrelin, and increased body mass index. *PLoS Med* 2004;1(3):e62. doi: 10.1371/journal.pmed.0010062 [published Online First: 2004/12/17]

7. Gottlieb DJ, Redline S, Nieto FJ, et al. Association of usual sleep duration with



For peer review only - http://bmjopen.bmj.com/site/about/guidelines.xhtml

hypertension: the Sleep Heart Health Study. *Sleep* 2006;29(8):1009-14. [published Online First: 2006/09/02]

8. Ayas NT, White DP, Manson JE, et al. A prospective study of sleep duration and coronary heart disease in women. *Arch Intern Med* 2003;163(2):205-9. [published Online First: 2003/01/28]

9. Kripke DF, Garfinkel L, Wingard DL, et al. Mortality associated with sleep duration and insomnia. *Arch Gen Psychiatry* 2002;59(2):131-6. [published Online First: 2002/02/05]

10. Kripke DF, Simons RN, Garfinkel L, et al. Short and long sleep and sleeping pills. Is increased mortality associated? *Arch Gen Psychiatry* 1979;36(1):103-16. [published Online First: 1979/01/01]

11. Lozano R, Naghavi M, Foreman K, et al. Global and regional mortality from 235 causes of death for 20 age groups in 1990 and 2010: a systematic analysis for the Global Burden of Disease Study 2010. *The Lancet* 2012;380(9859):2095-128. doi: http://dx.doi.org/10.1016/S0140-6736(12)61728-0

12. Third Report of the National Cholesterol Education Program (NCEP) Expert Panel on Detection, Evaluation, and Treatment of High Blood Cholesterol in Adults (Adult Treatment Panel III) final report. *Circulation* 2002;106(25):3143-421. [published Online First: 2002/12/18]

13. Schubert CM, Rogers NL, Remsberg KE, et al. Lipids, lipoproteins, lifestyle, adiposity



For peer review only - http://bmjopen.bmj.com/site/about/guidelines.xhtml

and fat-free mass during middle age: the Fels Longitudinal Study. *Int J Obes Relat Metab Disord* 2005;30(2):251-60.

14. Park Y-MM, Sui X, Liu J, et al. The Impact of Cardiorespiratory Fitness on Age-Related Lipids and Lipoproteins. *Journal of the American College of Cardiology* 2015;65(19):2091-100. doi: 10.1016/j.jacc.2015.03.517

15. Junqueira VBC, Barros SBM, Chan SS, et al. Aging and oxidative stress. *Molecular Aspects of Medicine* 2004;25(1–2):5-16. doi: https://doi.org/10.1016/j.mam.2004.02.003

16. Chua EC-P, Shui G, Cazenave-Gassiot A, et al. Changes in Plasma Lipids during Exposure to Total Sleep Deprivation. *Sleep* 2015;38(11):1683-91. doi: 10.5665/sleep.5142

17. Buysse DJ, Reynolds CF, III, Monk TH, et al. The Pittsburgh sleep quality index: A new instrument for psychiatric practice and research. *Psychiatry Research*;28(2):193-213. doi: 10.1016/0165-1781(89)90047-4

18. Choi KM, Lee JS, Park HS, et al. Relationship between sleep duration and the metabolic syndrome: Korean National Health and Nutrition Survey 2001. *Int J Obes (Lond)* 2008;32(7):1091-7. doi: 10.1038/ijo.2008.62 [published Online First: 2008/05/14]

19. Hall MH, Muldoon MF, Jennings JR, et al. Self-reported sleep duration is associated with the metabolic syndrome in midlife adults. *Sleep* 2008;31(5):635-43. [published Online First: 2008/06/04]

20. Bjorvatn B, Sagen IM, Oyane N, et al. The association between sleep duration, body mass

For peer review only



For peer review only - http://bmjopen.bmj.com/site/about/guidelines.xhtml

index and metabolic measures in the Hordaland Health Study. *J Sleep Res* 2007;16(1):66-76. doi: 10.1111/j.1365-2869.2007.00569.x [published Online First: 2007/02/21]

21. Williams CJ, Hu FB, Patel SR, et al. Sleep duration and snoring in relation to biomarkers of cardiovascular disease risk among women with type 2 diabetes. *Diabetes Care* 2007;30(5):1233-40. doi: 10.2337/dc06-2107 [published Online First: 2007/02/27]

22. Kaneita Y, Uchiyama M, Yoshiike N, et al. Associations of usual sleep duration with serum lipid and lipoprotein levels. *Sleep* 2008;31(5):645-52. [published Online First: 2008/06/04]

23. van den Berg JF, Miedema HM, Tulen JH, et al. Long sleep duration is associated with serum cholesterol in the elderly: the Rotterdam Study. *Psychosom Med* 2008;70(9):1005-11. doi: 10.1097/PSY.0b013e318186e656 [published Online First: 2008/10/10]

24. Manjareeka M, Nanda S, Mishra J, et al. Correlation between anthropometry and lipid profile in healthy subjects of Eastern India. *J Midlife Health* 2015;6(4):164-8. doi: 10.4103/0976-7800.172302 [published Online First: 2016/02/24]

25. Mota dos Santos C, Sa Silva C, Cesar de Araujo E, et al. [Lipid and glucose profiles in outpatients and their correlation with anthropometric indices]. *Rev Port Cardiol* 2013;32(1):35-41. doi: 10.1016/j.repc.2012.05.017 [published Online First: 2012/12/26]

For peer review only



For peer review only - http://bmjopen.bmj.com/site/about/guidelines.xhtml

26. Wu DM, Pai L, Sun PK, et al. Joint effects of alcohol consumption and cigarette smoking on atherogenic lipid and lipoprotein profiles: results from a study of Chinese male population in Taiwan. *Eur J Epidemiol* 2001;17(7):629-35. [published Online First: 2002/06/28]

27. Willett W, Hennekens CH, Castelli W, et al. Effects of cigarette smoking on fasting triglyceride, total cholesterol, and HDL-cholesterol in women. *Am Heart J* 1983;105(3):417-21. [published Online First: 1983/03/01]

28. De Oliveira e Silva ER, Foster D, McGee Harper M, et al. Alcohol Consumption Raises HDL Cholesterol Levels by Increasing the Transport Rate of Apolipoproteins A-I and A-II. *Circulation* 2000;102(19):2347-52. doi: 10.1161/01.cir.102.19.2347

29. Haskell WL. The influence of exercise on the concentrations of triglyceride and cholesterol in human plasma. *Exerc Sport Sci Rev* 1984;12:205-44. [published Online First: 1984/01/01]

30. Couillard C, Despres JP, Lamarche B, et al. Effects of endurance exercise training on plasma HDL cholesterol levels depend on levels of triglycerides: evidence from men of the Health, Risk Factors, Exercise Training and Genetics (HERITAGE) Family Study. *Arterioscler Thromb Vasc Biol* 2001;21(7):1226-32. [published Online First: 2001/07/14]

31. Hata Y, Nakajima K. Life-style and serum lipids and lipoproteins. *J Atheroscler Thromb* 2000;7(4):177-97. [published Online First: 2001/08/28]

For peer review only



For peer review only - http://bmjopen.bmj.com/site/about/guidelines.xhtml

32. Choudhury SR, Ueshima H, Kita Y, et al. Alcohol intake and serum lipids in a Japanese population. *Int J Epidemiol* 1994;23(5):940-7. [published Online First: 1994/10/01]

33. Spiegel K, Tasali E, Penev P, et al. Brief communication: Sleep curtailment in healthy young men is associated with decreased leptin levels, elevated ghrelin levels, and increased hunger and appetite. *Ann Intern Med* 2004;141(11):846-50. [published Online First: 2004/12/08]

34. Ayas NT, White DP, Al-Delaimy WK, et al. A prospective study of self-reported sleep duration and incident diabetes in women. *Diabetes Care* 2003;26(2):380-4. [published Online First: 2003/01/28]

35. Espiritu JR. Aging-related sleep changes. *Clin Geriatr Med* 2008;24(1):1-14, v. doi: 10.1016/j.cger.2007.08.007 [published Online First: 2007/11/24]

36. Edwards BA, O'Driscoll DM, Ali A, et al. Aging and sleep: physiology and pathophysiology. *Semin Respir Crit Care Med* 2010;31(5):618-33. doi: 10.1055/s-0030-1265902 [published Online First: 2010/10/14]

For peer review only



For peer review only - http://bmjopen.bmj.com/site/about/guidelines.xhtml

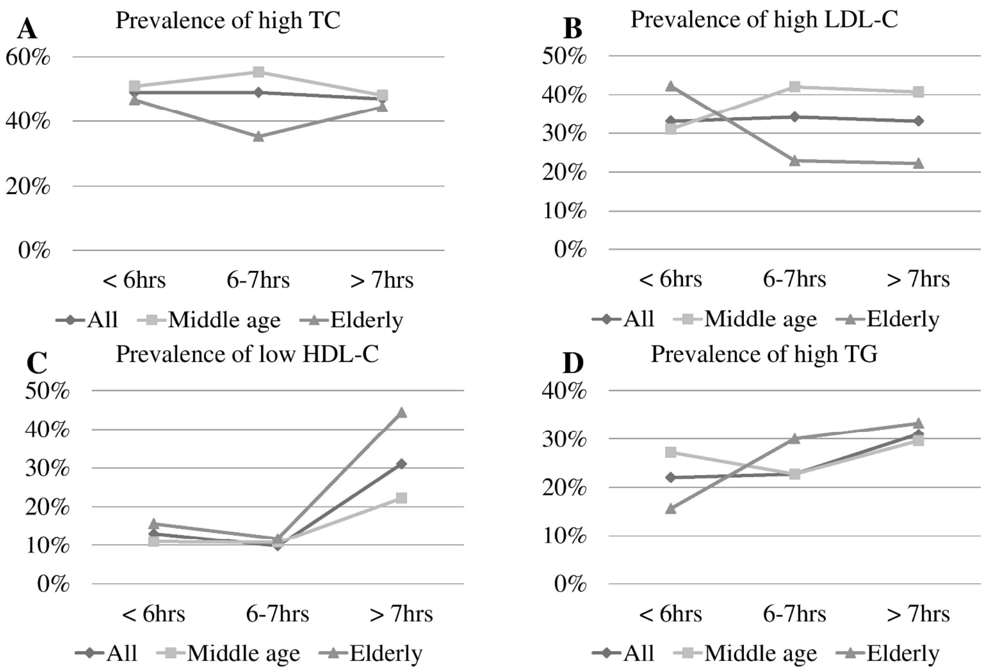


**Figure1.** Prevalence of serum lipid profiles by sleep duration between two age groups (middle age: 50-65 years old; elderly: ≥65 years old)

Figure1. Prevalence of serum lipid profiles by sleep duration between two age groups (middle age: 50-65 years old; elderly: ≥65 years old)

254x190mm (300 x 300 DPI)

ew only

**STROBE 2007 (v4) Statement—Checklist of items that should be included in reports of *cross-sectional studies***

| Section/Topic | Item # | Recommendation | Reported on page # |
|---|---|---|---|
| **Title and abstract** | 1 | (*a*) Indicate the study's design with a commonly used term in the title or the abstract | 1-3 |
| | | (*b*) Provide in the abstract an informative and balanced summary of what was done and what was found | 2-3 |
| **Introduction** | | | |
| Background/rationale | 2 | Explain the scientific background and rationale for the investigation being reported | 4-5 |
| Objectives | 3 | State specific objectives, including any prespecified hypotheses | 4-5 |
| **Methods** | | | |
| Study design | 4 | Present key elements of study design early in the paper | 5 |
| Setting | 5 | Describe the setting, locations, and relevant dates, including periods of recruitment, exposure, follow-up, and data collection | 5 |
| Participants | 6 | (*a*) Give the eligibility criteria, and the sources and methods of selection of participants | X |
| Variables | 7 | Clearly define all outcomes, exposures, predictors, potential confounders, and effect modifiers. Give diagnostic criteria, if applicable | 5-7 |
| Data sources/ measurement | 8* | For each variable of interest, give sources of data and details of methods of assessment (measurement). Describe comparability of assessment methods if there is more than one group | 5-6 |
| Bias | 9 | Describe any efforts to address potential sources of bias | *X* |
| Study size | 10 | Explain how the study size was arrived at | 5-6 |
| Quantitative variables | 11 | Explain how quantitative variables were handled in the analyses. If applicable, describe which groupings were chosen and why | 6-7 |
| Statistical methods | 12 | (*a*) Describe all statistical methods, including those used to control for confounding | 7 |
| | | (*b*) Describe any methods used to examine subgroups and interactions | X |
| | | (*c*) Explain how missing data were addressed | X |
| | | (*d*) If applicable, describe analytical methods taking account of sampling strategy | X |

| | | | |
|---|---|---|---|
| | | (*e*) Describe any sensitivity analyses | X |
| **Results** | | | |
| Participants | 13* | (a) Report numbers of individuals at each stage of study—eg numbers potentially eligible, examined for eligibility, confirmed eligible, included in the study, completing follow-up, and analysed | X |
| | | (b) Give reasons for non-participation at each stage | X |
| | | (c) Consider use of a flow diagram | X |
| Descriptive data | 14* | (a) Give characteristics of study participants (eg demographic, clinical, social) and information on exposures and potential confounders | 7-12 |
| | | (b) Indicate number of participants with missing data for each variable of interest | X |
| Outcome data | 15* | Report numbers of outcome events or summary measures | 7-14 |
| Main results | 16 | (*a*) Give unadjusted estimates and, if applicable, confounder-adjusted estimates and their precision (eg, 95% confidence interval). Make clear which confounders were adjusted for and why they were included | 13-14 |
| | | (*b*) Report category boundaries when continuous variables were categorized | X |
| | | (*c*) If relevant, consider translating estimates of relative risk into absolute risk for a meaningful time period | X |
| Other analyses | 17 | Report other analyses done—eg analyses of subgroups and interactions, and sensitivity analyses | X |
| **Discussion** | | | |
| Key results | 18 | Summarise key results with reference to study objectives | 15 |
| Limitations | 19 | Discuss limitations of the study, taking into account sources of potential bias or imprecision. Discuss both direction and magnitude of any potential bias | 17-18 |
| Interpretation | 20 | Give a cautious overall interpretation of results considering objectives, limitations, multiplicity of analyses, results from similar studies, and other relevant evidence | 15-18 |
| Generalisability | 21 | Discuss the generalisability (external validity) of the study results | X |
| **Other information** | | | |
| Funding | 22 | Give the source of funding and the role of the funders for the present study and, if applicable, for the original study on which the present article is based | 19 |

*Give information separately for cases and controls in case-control studies and, if applicable, for exposed and unexposed groups in cohort and cross-sectional studies.

**Note:** An Explanation and Elaboration article discusses each checklist item and gives methodological background and published examples of transparent reporting. The STROBE checklist is best used in conjunction with this article (freely available on the Web sites of PLoS Medicine at http://www.plosmedicine.org/, Annals of Internal Medicine at http://www.annals.org/, and Epidemiology at http://www.epidem.com/). Information on the STROBE Initiative is available at www.strobe-statement.org.

For peer review only

# Association between self-reported sleep duration and serum lipid profile in a middle-aged and elderly population in Taiwan: A community-based, cross-sectional study

| | |
|---|---|
| Journal: | *BMJ Open* |
| Manuscript ID | bmjopen-2017-015964.R2 |
| Article Type: | Research |
| Date Submitted by the Author: | 07-Sep-2017 |
| Complete List of Authors: | Lin, Pu; Chang Gung Memorial Hospital Linkou Branch, Family Medicine<br>Chang, Kai-Ting; Chang Gung Memorial Hospital Linkou Branch, Family Medicine<br>Lin, Yan-An; Chang Gung Memorial Hospital Linkou Branch, Family Medicine<br>Tzeng, I-Shiang; Taipei Tzu Chi General Hospital, Research<br>Chuang, Hai-Hua; Chang Gung Memorial Hospital Taipei Branch<br>Chen, Jau-Yuan; Chang Gung Memorial Hospital Linkou Branch, Family Medicine; Chang Gung University College of Medicine |
| <b>Primary Subject Heading</b>: | General practice / Family practice |
| Secondary Subject Heading: | Epidemiology, Health informatics |
| Keywords: | Cholesterol/Lipid, Family Health, Metabolism, Prevention, Sleep, Sleep Duration |
| | |

For peer review only

- **Article Title**

Association between self-reported sleep duration and serum lipid profile in a middle-aged and elderly population in Taiwan: A community-based, cross-sectional study

- **Running title**

Association between sleep duration and serum lipid profile

- **Author names and affiliations**

Pu Lin[1], Kai-Ting Chang[1], Yan-An Lin[1], I-Shiang Tzeng[2], Hai-Hua Chuang[3, #], Jau-Yuan Chen[1, 4, *]

[1]Department of Family Medicine, Chang-Gung Memorial Hospital, Linkou Branch, Taiwan

[2]Department of Research, Taipei Tzu Chi Hospital, Buddhist Tzu Chi Medical Foundation, New Taipei city, Taiwan

[3]Department of Family Medicine, Chang-Gung Memorial Hospital, Taipei Branch, Taiwan

[4]College of Medicine, Chang Gung University, Taoyuan, Taiwan

[#] Same contribution as the corresponding author

*Corresponding author

E-mail: welins@cgmh.org.tw (J.-Y. Chen)

Postal address: No. 5, Fuxing St., Guishan Dist., Taoyuan City 333, Taiwan (R.O.C.)

Phone: +886-975362672

Fax: +886-3-3287715

- **Format**

Word count: 2252

Number of black and white figures: 1



For peer review only - http://bmjopen.bmj.com/site/about/guidelines.xhtml

For peer review only

Number of Tables: 3

Number of References: 36

**ABSTRACT**

**Objectives:** The association between sleep duration and serum lipid profile in the middle-aged and the elderly is unclear. The aim of this study was to investigate and evaluate the relationships between sleep duration and levels of serum total cholesterol (TC), LDL cholesterol (LDL-C), HDL cholesterol (HDL-C), and triglycerides (TG) in these populations.

**Design:** Cross-sectional observational study.

**Setting:** Community-based investigation in Guishan Township of northern Taiwan.

**Participants:** A total of 400 community-dwelling middle-aged and elderly individuals were enrolled. All participants underwent a baseline assessment in 2014, which included anthropometrics, blood samples, and self-administered questionnaires. Participants were classified into three groups based on their sleep duration.

**Outcome measures:** Multivariate logistic regression was used to obtain odds ratios (ORs) and 95% confidence intervals (CIs) to assess the relationship between sleep duration and lipid profiles.

**Results:** Participant mean age was 64.5 years and 35.3% were men. Subjects with longer (>7 hours) and shorter (<6 hours) nightly sleep duration had a higher prevalence of low HDL-C levels (HDL <40 mg/dL) than those with moderate sleep duration (6-7 hours). Multivariate

For peer review only



For peer review only - http://bmjopen.bmj.com/site/about/guidelines.xhtml

logistic regression revealed that, compared to individuals with sleep duration of 6-7 hours, the odds of having low HDL-C were 3.68 (95% CI = 1.59-8.49) greater for individuals with sleep duration of <6 hours and 2.89 (95% CI = 1.10-7.61) greater for individuals with sleep duration of >7 hours.

**Conclusions:** There was a U-shaped relationship between sleep duration and HDL-C levels. Sleep duration over 7 hours or less than 6 hours increased the risk of low serum HDL-C levels.

**Keywords:** Cholesterol/Lipid, Family Health, Metabolism, Prevention, Sleep, Sleep Duration.

**Strengths and limitations of this study:**

■This is the first study to explore the associations between sleep duration and lipid profiles in middle-aged and elderly Taiwanese population.

■ We could not determine a causal relationship in our findings because this was a cross-sectional study.

■Lifestyle questionnaires were self-reported, which may limit the accuracy of measurements.

For peer review only

## INTRODUCTION

As known, most older adults experience lipid profile disorders.[1] Moreover, the different levels in the percentage of lipid disorders may have an association with demographic factors (such as age and sex).[1] A study also found a significant association between the high prevalence of dyslipidemia and risk factors, such as increasing age, smoking status, hypertension, diabetes, and body mass index.[2] Several studies have identified that longer or shorter sleep duration may increase the mortality and morbidity risks from diabetes mellitus,[3-5] obesity,[6] hypertension,[7] and coronary heart disease.[8] Some studies have shown a U-shaped association between sleep duration and major morbidities.[5 9 10] Cardiovascular disease has been a leading cause of death worldwide.[11] Dyslipidemia, such as high levels of total cholesterol (TC), low-density lipoprotein cholesterol (LDL-C), and triglycerides (TG) and low levels of high-density lipoprotein cholesterol (HDL-C), increases the risk of cardiovascular disease,[12] and hence remains an important issue in the field of health promotion and disease prevention. Previous studies have shown age-associated alterations in the level, composition, and function of lipid profiles, including an inverted U-shaped quadratic trajectory for TC, LDL-C, and TG,[13 14] and a decrease in anti-oxidative ability.[15] Other than age, factors that have been suggested to be related to lipid profile levels include body mass index (BMI), body composition, diet, and cardiorespiratory fitness.[13 14]



For peer review only - http://bmjopen.bmj.com/site/about/guidelines.xhtml

Although some studies suggest that sleep deprivation may result in changes to plasma lipid levels,[16] there is a lack of consensus on the possible associations that may exist between sleep duration and serum lipid profiles, and even fewer data are available in the Taiwanese population.[17] Therefore, we investigated the relationship between sleep duration and levels of serum TC, LDL-C, HDL-C, and TG in a community-based study in Taiwan.

## MATERIALS AND METHODS

### Study participants

The present study was an observational, cross-sectional study. We enrolled 400 participants, including 141 men and 259 women. The inclusion criteria included (1) residents aged over 50 years, and (2) the residents living in Guishan Township. Twelve people aged younger than 50 years old were excluded from the study. Subjects were excluded if (1) they could not complete the full examinations or had missing data for age, sex, anthropometric values and blood test results; (2) were functionally dependent; (3) were unable to adequately communicate with the interviewers; (4) had major illnesses recently; or (5) had known sleep disorders.

### Data collection

Data collection comprised two parts: a physical status examination and a self-administered questionnaire. For the physical status examination, height, weight,



For peer review only - http://bmjopen.bmj.com/site/about/guidelines.xhtml

abdominal circumference, blood pressure, and blood samples were collected for all participants. Blood samples included lipid profiles (TC, LDL-C, HDL-C, and TG), aspartate aminotransferase (AST) levels, fasting plasma glucose (FPG) levels, creatinine levels, and uric acid levels. The self-administered questionnaire included items related to smoking, drinking, exercise, sleep, and other lifestyle habits. Details from all the participants were obtained on the same day while the data collection was performed from March to August 2014.

**Definitions and variables**

Based on the National Cholesterol Education Program (NCEP) Adult Treatment Panel (ATP) III guidelines, we set different cutoff points for each item of the serum lipid profiles (LDL-C: 130 mg/dL; TC: 200 mg/dL; HDL-C: 40 mg/dL; and TG: 150 mg/dL). Hyperlipidemia was defined as having LDL-C, TC, or TG levels above the cutoff points.

Adapted from the Pittsburgh Sleep Quality Index (PSQI),[17] the questionnaire about sleep included the question: "What was your daily average sleep duration during the past month (not counting the times lying on the bed without sleeping)?". We divided sleep duration into three groups: less than 6 hours (<6 hours), greater than or equal to 6 hours but less than or equal to 7 hours (6-7 hours), and more than 7 hours (>7 hours). Hypertension was defined as a self-reported hypertension or the use of anti-hypertensive drugs. Diabetes was defined as a self-reported diabetes mellitus or the use of oral anti-diabetic drugs or insulin. Alcohol



For peer review only - http://bmjopen.bmj.com/site/about/guidelines.xhtml

drinking was defined as drinking alcohol on greater than or equal to 2 days per week. Regular exercise was defined as exercising greater or equal to 2 days per week. Current smoking was defined as the literal meaning.

The institutional review board (IRB) approved the study and all patients provided written informed consent.

**Statistical analysis**

Adequate blood samples and complete questionnaires were collected from all participants. After data were collected, statistical analysis was conducted using the SPSS 21.0 statistical package (SPSS Inc., Chicago, USA). A $p$-value <0.05 was considered statistically significant. Our statistical analysis included multiple steps. First, characteristics were compared across hyperlipidemia and sleep duration sub-groups using t-tests, one-way ANOVA, and chi-square tests. Homogeneity examination was performed with ANOVA, and heterogeneous variables were analyzed with the Brown-Forsythe test. Second, we measured correlations between serum lipid profiles and related risk factors. Third, logistic regression analyses were conducted to assess the relationship between sleep duration and the risk of high LDL-C, high TC, low HDL-C, and high TG levels; additional regression models were adjusted for relevant covariates.

**RESULTS**

The characteristics of study subjects with and without hyperlipidemia are shown in Table



For peer review only - http://bmjopen.bmj.com/site/about/guidelines.xhtml

1. Among the included participants, 260 (65%) had hyperlipidemia. Body mass index, waist circumference, waist-to-height ratio, AST levels, FPG levels, and uric acid levels were significantly higher in the hyperlipidemia group.

For peer review only



For peer review only - http://bmjopen.bmj.com/site/about/guidelines.xhtml

78 **Table 1** Characteristics of the study subjects with and without hyperlipidemia.

| Variables | No hyperlipidemia (n=140) | Hyperlipidemia (n=260) | *p*-value |
|---|---|---|---|
| Sex (male) | 57 (40.7) | 84 (32.3) | 0.101 |
| Age (years) | 65.4 ± 8.4 | 64.0 ± 8.4 | 0.072 |
| Current smoking | 9 (6.4) | 34 (13.1) | 0.043 |
| Alcohol drinking | 20 (14.3) | 55 (21.2) | 0.107 |
| Regular exercise | 116 (83.2) | 211 (81.2) | 0.681 |
| Hypertension | 64 (45.7) | 137 (52.7) | 0.209 |
| Diabetes | 28 (19.7) | 56 (21.5) | 0.238 |
| LDL-C (mg/dL) | 97.6 ± 17.7 | 129.6 ± 32.6 | <0.001 |
| TC (mg/dL) | 169.8 ± 21.1 | 211.9 ± 33.2 | <0.001 |
| HDL-C (mg/dL) | 55.4 ± 12.8 | 53.9 ± 14.5 | 0.161 |
| TG (mg/dL) | 84.5 ± 29.3 | 142.3 ± 71.6 | <0.001 |
| Sleep (hours) | 6.2 ± 1.2 | 6.2 ± 1.3 | 0.276 |
| SBP (mmHg) | 128.0 ± 16.0 | 130.2 ± 17.0 | 0.267 |
| DBP (mmHg) | 76.1 ± 11.3 | 77.1 ± 12.2 | 0.592 |
| BMI (kg/m$^2$) | 23.7 ± 3.4 | 25.0 ± 3.6 | 0.001 |
| WC (cm) | 82.9 ± 8.5 | 86.3 ± 10.1 | 0.001 |
| WHtR | 0.52 ± 0.05 | 0.54 ± 0.06 | 0.003 |
| ALT (mg/dL) | 20.3 ± 8.9 | 23.9 ± 14.6 | 0.032 |
| Creatinine (mg/dL) | 0.8 ± 0.6 | 0.8 ± 0.3 | 0.408 |
| FPG (mg/dL) | 92.2 ± 19.1 | 98.4 ± 28.5 | 0.028 |
| Uric acid (mg/dL) | 5.4 ± 1.3 | 5.9 ± 1.4 | 0.001 |

79 Data expressed as mean ± SD for continuous variables and n (%) for categorical variables.
80 Abbreviations: TC = total cholesterol; TG = triglyceride; LDL-C = low-density lipoprotein;
81 HDL-C = high-density lipoprotein; Sleep = sleep duration; ALT = alanine transaminase; WC
82 = waist circumference; WHtR = waist to height ratio; BMI = body mass index; FPG = fasting
83 plasma glucose; SBP = systolic blood pressure; DBP = diastolic blood pressure.

For peer review only



For peer review only - http://bmjopen.bmj.com/site/about/guidelines.xhtml

The characteristics of the study subjects categorized by sleep duration are shown in Table 2. There were 100, 255, and 45 participants in the <6 hours, 6-7 hours, and the >7 hours groups respectively. Low HDL-C levels were significantly higher in the >7 hours group (31.1%) than in the other two groups (9.8% for the 6-7 hours group and 13.0% for the <6 hours group). The post-hoc tests also showed that the difference was significant (significant difference between <6 hours and >7 hours groups ($p$ value = 0.009), 6-7 hours and >7 hours (p value=0.001).

For peer review only



For peer review only - http://bmjopen.bmj.com/site/about/guidelines.xhtml

**Table 2** Characteristics of the study subjects by sleep duration groups.

| Variable | <6 hours (n = 100) | 6-7 hours (n = 255) | >7 hours (n = 45) | *p*-value |
|---|---|---|---|---|
| Sex (male) | 30 (30) | 88 (34.5) | 23 (51.1)*† | 0.044 |
| Age (years) | 65.2 ± 7.6 | 64.1 ± 8.3 | 65.1 ± 10.6 | 0.530 |
| LDL-C (mg/dL) | 115.8 ± 30.4 | 119.2 ± 31.3 | 119.6 ± 40.0 | 0.643 |
| TC (mg/dL) | 197.2 ± 34.9 | 197.0 ± 34.2 | 198.0 ± 45.4 | 0.984 |
| HDL-C (mg/dL) | 57.4 ± 15.8 | 53.9 ± 12.5 | 51.2 ± 16.4 | 0.025 |
| TG (mg/dL) | 120.4 ± 61.8 | 119.8 ± 62.7 | 138.5 ± 88.6 | 0.207 |
| High LDL-C | 33 (33) | 87 (34.1) | 15 (33.3) | 0.978 |
| High TC | 49 (49) | 125 (49.0) | 21 (47.7) | 0.941 |
| Low HDL-C | 13 (13) | 25 (9.8) | 14 (31.1)*† | 0.001 |
| High TG | 22 (22) | 58 (22.7) | 14 (31.1) | 0.437 |
| Alcohol drinking | 15 (15) | 52 (20.4) | 11 (24.4) | 0.346 |
| Current Smoking | 10 (10) | 27 (10.6) | 6 (13.3) | 0.828 |
| Regular exercise | 80 (80) | 209 (81.9) | 39 (86.4) | 0.642 |
| Hypertension | 47 (47) | 125 (49.0) | 29 (64.4) | 0.122 |
| Diabetes | 25 (25) | 45 (17.6) | 9 (20.0) | 0.293 |
| Hyperlipidemia | 69 (69) | 161 (63.1) | 30 (66.7) | 0.564 |
| SBP (mmHg) | 129.9 ± 16.5 | 128.6 ± 16.7 | 133.3 ± 25.2 | 0.216 |
| DBP (mmHg) | 76.4 ± 13.0 | 76.6 ± 11.8 | 78.9 ± 9.13 | 0.436 |
| BMI (kg/m$^2$) | 24.3 ± 3.9 | 24.6 ± 3.4 | 25.1 ± 3.6 | 0.394 |
| WC (cm) | 84.7 ± 10.4 | 84.8 ± 9.6 | 87.4 ± 8.6 | 0.235 |
| WHtR | 0.5 ± 0.1 | 0.6 ± 0.1 | 0.6 ± 0.1 | 0.417 |
| ALT (mg/dL) | 22.5 ± 14.0 | 22.3 ± 11.94 | 22.7 ± 15.9 | 0.530 |
| Creatinine (mg/dL) | 0.8 ± 0.3 | 0.8 ± 0.3 | 1.0 ± 0.9 | 0.920 |
| FPG (mg/dL) | 98.2 ± 28.2 | 94.8 ± 19.3 | 100.3 ± 45.1 | 0.519 |
| Uric acid (mg/dL) | 5.6 ± 1.3 | 5.8 ± 1.4 | 6.1 ± 1.4 | 0.101 |

Data expressed as mean ± SD for continuous variables and n (%) for categorical variables. * *p*-value < 0.05 compared with <6 hours group, † p-value < 0.05 compared with 6-7 hours group.

Abbreviations: High TC = total cholesterol ≥200 (mg/d); High TG = triglyceride ≥150 (mg/d); High LDL-C = low-density lipoprotein ≥130 (mg/d); Low HDL-C = high-density lipoprotein ≤40 (mg/d); ALT = alanine transaminase; WC = waist circumference; WHtR =

For peer review only



For peer review only - http://bmjopen.bmj.com/site/about/guidelines.xhtml

waist to height ratio; BMI = body mass index; FPG = fasting plasma glucose; SBP = systolic blood pressure; DBP = diastolic blood pressure.

For peer review only



For peer review only - http://bmjopen.bmj.com/site/about/guidelines.xhtml

Figures 1 shows the comparisons of serum lipid profiles by sleep duration for two age groups: equal to or greater than 65 years (elderly group, 168 people) and all others (middle age group, 232 people). Abnormal total cholesterol prevalence levels were the highest (around 50%) among the four items with no significant differences by sleep duration (Figure 1A). The average prevalence of high LDL-C was about one-third, and the prevalence gradually increased with longer sleep duration in the middle age group but decreased with longer sleep duration in the elderly group (Figure 1B). There was a U-shaped (or J-shaped) distribution in the association of low HDL-C levels with sleep duration with both age groups showing significantly higher prevalence of low HDL-C when the sleep duration was <6 hours or >7 hours (Figure 1C). Abnormal triglyceride levels by sleep duration in the middle age group also appeared to have a U-shaped distribution (Figure 1D).

For peer review only



For peer review only - http://bmjopen.bmj.com/site/about/guidelines.xhtml

Multivariable logistic regression model results are shown in Table 3. Model 1 assessed the crude odds ratio of lipid profiles by sleep duration; Model 2 adjusted for age; Model 3 adjusted for age and waist circumference; and Model 4 adjusted for age, waist circumference, and other traditional factors that influence lipid levels, including sex, alcohol drinking, exercise, and smoking. The 6–7-hour sleep duration group was set as the reference category. In a model where high TC levels, high LDL-C levels, or high TG levels were the outcome, no significant association was found with sleep duration. However, significant association was found in Models 1 through 4 when the dependent variable was low HDL-C levels (Table 3).



For peer review only - http://bmjopen.bmj.com/site/about/guidelines.xhtml

120 **Table 3** Odds ratios for the associate between lipid profiles and reported sleep duration.

| | **Sleep <6 hours (n=100)** | **Sleep 6-7 hours (n=255)** | **Sleep >7 hours (n=45)** |
|---|---|---|---|
| | Total cholesterol | | |
| Model 1 | 1.02 (0.54-1.92) | 1.00 | 1.10 (0.54-2.22) |
| Model 2 | 0.99 (0.52-1.88) | 1.00 | 1.10 (0.52-2.24) |
| Model 3 | 1.01 (0.53-1.93) | 1.00 | 1.13 (0.56-2.31) |
| Model 4 | 0.90 (0.45-1.80) | 1.00 | 0.95 (0.44-2.02) |
| | LDL-C | | |
| Model 1 | 1.04 (0.53-2.03) | 1.00 | 0.99 (0.47-2.08) |
| Model 2 | 1.02 (0.52-2.00) | 1.00 | 0.99 (0.47-2.10) |
| Model 3 | 1.05 (0.53-2.07) | 1.00 | 1.03 (0.49-2.19) |
| Model 4 | 1.001(0.50-2.01) | 1.00 | 0.94 (0.43-2.04) |
| | HDL-C | | |
| Model 1 | 3.81 (1.80-8.05)* | 1.00 | 3.02 (1.28-7.14)* |
| Model 2 | 3.75 (1.77-7.93)* | 1.00 | 3.02 (1.28-7.16)* |
| Model 3 | 3.62 (1.63-8.00)* | 1.00 | 2.93 (1.17-7.34)* |
| Model 4 | 3.68 (1.59-8.49)* | 1.00 | 2.89 (1.10-7.61)* |
| | Triglyceride | | |
| Model 1 | 0.65 (0.33-1.31) | 1.00 | 0.63 (0.28-1.37) |
| Model 2 | 0.65 (0.33-1.31) | 1.00 | 0.63 (0.27-1.38) |
| Model 3 | 0.72 (0.35-1.46) | 1.00 | 0.69 (0.31-1.35) |
| Model 4 | 0.64 (0.31-1.34) | 1.00 | 0.60 (0.26-1.39) |

121 Data are expressed as odds ratio (OR) and 95% confidence interval (CI))

122 *P-value <0.05

123 †Model 1: unadjusted; Model 2: multiple logistic regression adjusted for age; Model 3:
124 multiple logistic regression adjusted for age and waist circumference; Model 4: multiple
125 logistic regression adjusted for age, sex, waist circumference, alcohol drinking, exercise, and
126 smoking.

127 Abbreviations: TC=total cholesterol; TG=triglyceride; LDL-C=low-density lipoprotein;
128 HDL-C= high-density lipoprotein.



For peer review only - http://bmjopen.bmj.com/site/about/guidelines.xhtml

# DISCUSSION

In our study, traditional risk factors, such as high BMI, high waist circumference, high FPG levels, and high uric acid levels, were significantly associated with abnormal lipid values. Elevated AST levels may be associated with non-alcoholic liver disease. We found a U-shaped association between sleep duration and low HDL-C levels, and logistic regression models showed that sleep duration was significantly associated with low HDL-C levels regardless of the adjustment for potential confounders.

Several studies have reported the associations between serum lipid profiles and sleep duration, but the results of these studies have been inconsistent.[18-23] Choi et al.[18] collected data from 4,222 Korean participants over the age of 60 years and found a U-shaped association between low HDL-C levels and high triglyceride levels, which were similar to the results of our study. Both short and long sleep durations were related to an increased risk of metabolic syndrome and sleep duration of 7 hours demonstrated the lowest prevalence of metabolic syndrome in this study. Hall et al.[19] reported that sleep duration was independently associated with three components of metabolic syndrome: abdominal obesity, elevated serum glucose levels, and elevated triglyceride levels. The optimal sleep duration for preventing metabolic syndrome determined by this study was 7 to 8 hours per night. Bjorvatn et al.[20] demonstrated that cholesterol levels, triglyceride levels, and blood pressure were higher in subjects with short sleep duration. Another study[21] revealed that HDL-C levels decreased with



For peer review only - http://bmjopen.bmj.com/site/about/guidelines.xhtml

short and long sleep duration among normotensive, but not hypertensive, women.

The logistic regression models, adjusted for age, sex, waist circumference, alcohol drinking, exercise, and smoking, showed significant association with low HDL-C levels. To our knowledge, the prevalence of dyslipidemia is proportional to age and different in men and women. The association between waist circumference and dyslipidemia has been confirmed in some studies.[24 25] Lifestyle factors have been shown to influence serum lipid or lipoprotein levels. For example, smoking decreases HDL-C levels and increases TG levels, whereas alcohol consumption increases the levels of both.[26-28] Exercise increases HDL-C levels and decreases TG levels.[29 30] In addition, alcohol consumption is reported to decrease LDL-C levels.[31 32] Even after adjusting for these potential confounding factors, our logistic regression models showed significant differences in odds of low HDL-C levels between sleep duration groups.

The influence that shorter sleep duration has on body weight and dyslipidemia has become clearer in recent years. Sleep restriction is associated with hormone imbalance; it reduces leptin (an appetite suppressant) and elevates ghrelin levels (an appetite stimulant),[6 33] which may contribute to increased body weight and lead to dyslipidemia. The biochemical mechanism for the relationship between longer sleep and dyslipidemia has not been clearly confirmed; some studies showed that prolonged sleep duration may be associated with glucose intolerance and diabetes.[3 34] Physical performance, such as reduced energy



For peer review only - http://bmjopen.bmj.com/site/about/guidelines.xhtml

consumption due to increased time in bed, may affect obesity; one study explained this relationship by showing that longer sleep duration was related to less exercise.[8]

Some studies have reported that the relative risk of mortality and morbidity was the lowest when sleep duration was 7-8 hours per night,[5 8-10] but other research has reported that 6-7 hours is more optimal.[22] The cutoff point of our study was set at 6-7 hours; we found the volunteers in our study generally woke up early regardless of the amount of time they slept, and they often had spontaneous arousal from nocturnal sleep. This may be explained by physiological age-related changes in circadian modulation, homeostatic factors, cardiopulmonary function, and endocrine function;[35 36] or be due to underlying chronic diseases. In an attempt to recommend the optimal sleep duration for lowering morbidity based on epidemiological data, it is inferred that the optimal sleep duration will vary for different populations.[22]

Limitations of this study should be mentioned. First, since this was a cross-sectional study, a cohort study is required for determining more causal relationships. Second, selection bias might exist because the volunteer participant selection was limited to one township, and participants were not selected randomly or with a stratified method from the population. This could limit the generalizability of these results. In addition, our sample size was relatively small; hence, sampling bias should be considered. Third, we did not group the population by underlying diseases, such as diabetes or cerebrovascular disease, due to the limited sample

For peer review only



For peer review only - http://bmjopen.bmj.com/site/about/guidelines.xhtml

size. The proper cutoff for lipid profiles may differ according to underlying diseases. Furthermore, the HDL-C cutoff point differs by sex; in metabolic syndrome, it is set as 40 mg/dL for men and 50 mg/dL for women. The influence of sleep duration on lipid metabolism may also differ between men and women; in a study by Kaneita et al.,[22] among 1,666 men and 2,329 women aged 20 years or older from Japan, it was shown that HDL-C levels had a U-shaped association with sleep duration in women but not in men. Fourth, glycosylated hemoglobin might be a more appropriate measurement of diabetes instead of self-reported diabetes mellitus or the use of oral anti-diabetic drugs or insulin. Fifth, similar to the majority of studies in the literature examining the effects of sleep duration on serum lipids, diet was not factored in as a covariate. It may be explained that daily life diet is a diversified condition. Thus, it may be difficult to represent it with just one variable in order to adjust for it in the regression model. Sixth, lipid-lowering therapy was an important factor influencing lipid levels, but we did not consider lipid-lowering medications while formulating the questionnaire. Finally, the present study estimated sleep duration only by a self-reported questionnaire, which is a more systematically biased estimate than measured sleep duration.

## CONCLUSIONS

In our study of a Taiwanese population, sleep duration of 6-7 hours per night reduced the risk of abnormal serum lipid profiles. Sleep duration over 7 hours or less than 6 hours may increase the risk of low serum HDL-C levels, although adjustment for risk factors attenuated



For peer review only - http://bmjopen.bmj.com/site/about/guidelines.xhtml

this relationship. Inappropriate sleep duration might be a potential risk factor for low HDL-C levels, and adequate sleep duration may improve low HDL-C status. Lifestyle interventions, including exercise and abstaining from alcohol and smoking, should be initiated early in high-risk groups.

For peer review only



For peer review only - http://bmjopen.bmj.com/site/about/guidelines.xhtml

**Contributors**

PL was involved in the data collection, analysis, and writing of the manuscript. KTC and YAL were involved in the collection of data. IST provided statistical advice. HHC and JYC contributed to the conceptualization; they designed, performed the experiments, collected and analyzed the data, as well as revising it critically for important intellectual content; and also approved the final version to be submitted.

**Funding** This work was supported by Chang Gung Memorial Hospital (CORPG3C0011, CORPG3C0171, CORPG3C0172, CZRPG3C0053).

**Competing interests** None declared.

**Ethics approval** The study was approved by Chang-Gung Medical Foundation Institutional Review Board (102-2304B), and written informed consent was given by all the participants before enrollment.

**Data sharing statement** No additional data are available.

For peer review only



For peer review only - http://bmjopen.bmj.com/site/about/guidelines.xhtml

## REFERENCES

1. Hoffmann K, Matyjaszczyk P, Zaporowska-Stachowiak I, et al. Lipid profile of polish primary care patients aged 35-55 years living in the district of Pleszew. *Acta Poloniae Pharmaceutica* 2016;73(2):537-46.

2. Ni W-Q, Liu X-L, Zhuo Z-P, et al. Serum lipids and associated factors of dyslipidemia in the adult population in Shenzhen. *Lipids in Health and Disease* 2015;14:71. doi: 10.1186/s12944-015-0073-7

3. Gottlieb DJ, Punjabi NM, Newman AB, et al. Association of sleep time with diabetes mellitus and impaired glucose tolerance. *Arch Intern Med* 2005;165(8):863-7. doi: 10.1001/archinte.165.8.863 [published Online First: 2005/04/27]

4. Yaggi HK, Araujo AB, McKinlay JB. Sleep duration as a risk factor for the development of type 2 diabetes. *Diabetes Care* 2006;29(3):657-61. [published Online First: 2006/03/01]

5. Tamakoshi A, Ohno Y. Self-reported sleep duration as a predictor of all-cause mortality: results from the JACC study, Japan. *Sleep* 2004;27(1):51-4. [published Online First: 2004/03/05]

6. Taheri S, Lin L, Austin D, et al. Short sleep duration is associated with reduced leptin, elevated ghrelin, and increased body mass index. *PLoS Med* 2004;1(3):e62. doi: 10.1371/journal.pmed.0010062 [published Online First: 2004/12/17]

7. Gottlieb DJ, Redline S, Nieto FJ, et al. Association of usual sleep duration with



For peer review only - http://bmjopen.bmj.com/site/about/guidelines.xhtml

hypertension: the Sleep Heart Health Study. *Sleep* 2006;29(8):1009-14. [published Online First: 2006/09/02]

8. Ayas NT, White DP, Manson JE, et al. A prospective study of sleep duration and coronary heart disease in women. *Arch Intern Med* 2003;163(2):205-9. [published Online First: 2003/01/28]

9. Kripke DF, Garfinkel L, Wingard DL, et al. Mortality associated with sleep duration and insomnia. *Arch Gen Psychiatry* 2002;59(2):131-6. [published Online First: 2002/02/05]

10. Kripke DF, Simons RN, Garfinkel L, et al. Short and long sleep and sleeping pills. Is increased mortality associated? *Arch Gen Psychiatry* 1979;36(1):103-16. [published Online First: 1979/01/01]

11. Lozano R, Naghavi M, Foreman K, et al. Global and regional mortality from 235 causes of death for 20 age groups in 1990 and 2010: a systematic analysis for the Global Burden of Disease Study 2010. *The Lancet* 2012;380(9859):2095-128. doi: http://dx.doi.org/10.1016/S0140-6736(12)61728-0

12. Third Report of the National Cholesterol Education Program (NCEP) Expert Panel on Detection, Evaluation, and Treatment of High Blood Cholesterol in Adults (Adult Treatment Panel III) final report. *Circulation* 2002;106(25):3143-421. [published Online First: 2002/12/18]

13. Schubert CM, Rogers NL, Remsberg KE, et al. Lipids, lipoproteins, lifestyle, adiposity

For peer review only



For peer review only - http://bmjopen.bmj.com/site/about/guidelines.xhtml

and fat-free mass during middle age: the Fels Longitudinal Study. *Int J Obes Relat Metab Disord* 2005;30(2):251-60.

14. Park Y-MM, Sui X, Liu J, et al. The Impact of Cardiorespiratory Fitness on Age-Related Lipids and Lipoproteins. *Journal of the American College of Cardiology* 2015;65(19):2091-100. doi: 10.1016/j.jacc.2015.03.517

15. Junqueira VBC, Barros SBM, Chan SS, et al. Aging and oxidative stress. *Molecular Aspects of Medicine* 2004;25(1–2):5-16. doi: https://doi.org/10.1016/j.mam.2004.02.003

16. Chua EC-P, Shui G, Cazenave-Gassiot A, et al. Changes in plasma lipids during exposure to total sleep deprivation. *Sleep* 2015;38(11):1683-91. doi: 10.5665/sleep.5142

17. Buysse DJ, Reynolds CF, III, Monk TH, et al. The Pittsburgh sleep quality index: A new instrument for psychiatric practice and research. *Psychiatry Research*;28(2):193-213. doi: 10.1016/0165-1781(89)90047-4

18. Choi KM, Lee JS, Park HS, et al. Relationship between sleep duration and the metabolic syndrome: Korean National Health and Nutrition Survey 2001. *Int J Obes (Lond)* 2008;32(7):1091-7. doi: 10.1038/ijo.2008.62 [published Online First: 2008/05/14]

19. Hall MH, Muldoon MF, Jennings JR, et al. Self-reported sleep duration is associated with the metabolic syndrome in midlife adults. *Sleep* 2008;31(5):635-43. [published Online First: 2008/06/04]

20. Bjorvatn B, Sagen IM, Oyane N, et al. The association between sleep duration, body mass

For peer review only



For peer review only - http://bmjopen.bmj.com/site/about/guidelines.xhtml

index and metabolic measures in the Hordaland Health Study. *J Sleep Res* 2007;16(1):66-76. doi: 10.1111/j.1365-2869.2007.00569.x [published Online First: 2007/02/21]

21. Williams CJ, Hu FB, Patel SR, et al. Sleep duration and snoring in relation to biomarkers of cardiovascular disease risk among women with type 2 diabetes. *Diabetes Care* 2007;30(5):1233-40. doi: 10.2337/dc06-2107 [published Online First: 2007/02/27]

22. Kaneita Y, Uchiyama M, Yoshiike N, et al. Associations of usual sleep duration with serum lipid and lipoprotein levels. *Sleep* 2008;31(5):645-52. [published Online First: 2008/06/04]

23. van den Berg JF, Miedema HM, Tulen JH, et al. Long sleep duration is associated with serum cholesterol in the elderly: the Rotterdam Study. *Psychosom Med* 2008;70(9):1005-11. doi: 10.1097/PSY.0b013e318186e656 [published Online First: 2008/10/10]

24. Manjareeka M, Nanda S, Mishra J, et al. Correlation between anthropometry and lipid profile in healthy subjects of Eastern India. *J Midlife Health* 2015;6(4):164-8. doi: 10.4103/0976-7800.172302 [published Online First: 2016/02/24]

25. Mota dos Santos C, Sa Silva C, Cesar de Araujo E, et al. [Lipid and glucose profiles in outpatients and their correlation with anthropometric indices]. *Rev Port Cardiol* 2013;32(1):35-41. doi: 10.1016/j.repc.2012.05.017 [published Online First: 2012/12/26]

For peer review only



For peer review only - http://bmjopen.bmj.com/site/about/guidelines.xhtml

26. Wu DM, Pai L, Sun PK, et al. Joint effects of alcohol consumption and cigarette smoking on atherogenic lipid and lipoprotein profiles: results from a study of Chinese male population in Taiwan. *Eur J Epidemiol* 2001;17(7):629-35. [published Online First: 2002/06/28]

27. Willett W, Hennekens CH, Castelli W, et al. Effects of cigarette smoking on fasting triglyceride, total cholesterol, and HDL-cholesterol in women. *Am Heart J* 1983;105(3):417-21. [published Online First: 1983/03/01]

28. De Oliveira e Silva ER, Foster D, McGee Harper M, et al. Alcohol Consumption Raises HDL Cholesterol Levels by Increasing the Transport Rate of Apolipoproteins A-I and A-II. *Circulation* 2000;102(19):2347-52. doi: 10.1161/01.cir.102.19.2347

29. Haskell WL. The influence of exercise on the concentrations of triglyceride and cholesterol in human plasma. *Exerc Sport Sci Rev* 1984;12:205-44. [published Online First: 1984/01/01]

30. Couillard C, Despres JP, Lamarche B, et al. Effects of endurance exercise training on plasma HDL cholesterol levels depend on levels of triglycerides: evidence from men of the Health, Risk Factors, Exercise Training and Genetics (HERITAGE) Family Study. *Arterioscler Thromb Vasc Biol* 2001;21(7):1226-32. [published Online First: 2001/07/14]

31. Hata Y, Nakajima K. Life-style and serum lipids and lipoproteins. *J Atheroscler Thromb* 2000;7(4):177-97. [published Online First: 2001/08/28]

For peer review only



For peer review only - http://bmjopen.bmj.com/site/about/guidelines.xhtml

32. Choudhury SR, Ueshima H, Kita Y, et al. Alcohol intake and serum lipids in a Japanese population. *Int J Epidemiol* 1994;23(5):940-7. [published Online First: 1994/10/01]

33. Spiegel K, Tasali E, Penev P, et al. Brief communication: Sleep curtailment in healthy young men is associated with decreased leptin levels, elevated ghrelin levels, and increased hunger and appetite. *Ann Intern Med* 2004;141(11):846-50. [published Online First: 2004/12/08]

34. Ayas NT, White DP, Al-Delaimy WK, et al. A prospective study of self-reported sleep duration and incident diabetes in women. *Diabetes Care* 2003;26(2):380-4. [published Online First: 2003/01/28]

35. Espiritu JR. Aging-related sleep changes. *Clin Geriatr Med* 2008;24(1):1-14, v. doi: 10.1016/j.cger.2007.08.007 [published Online First: 2007/11/24]

36. Edwards BA, O'Driscoll DM, Ali A, et al. Aging and sleep: physiology and pathophysiology. *Semin Respir Crit Care Med* 2010;31(5):618-33. doi: 10.1055/s-0030-1265902 [published Online First: 2010/10/14]

**Figure Legends**

Figure1. Prevalence of serum lipid profiles by sleep duration between two age groups (middle age: 50-65 years old; elderly: ≥65 years old)



For peer review only - http://bmjopen.bmj.com/site/about/guidelines.xhtml

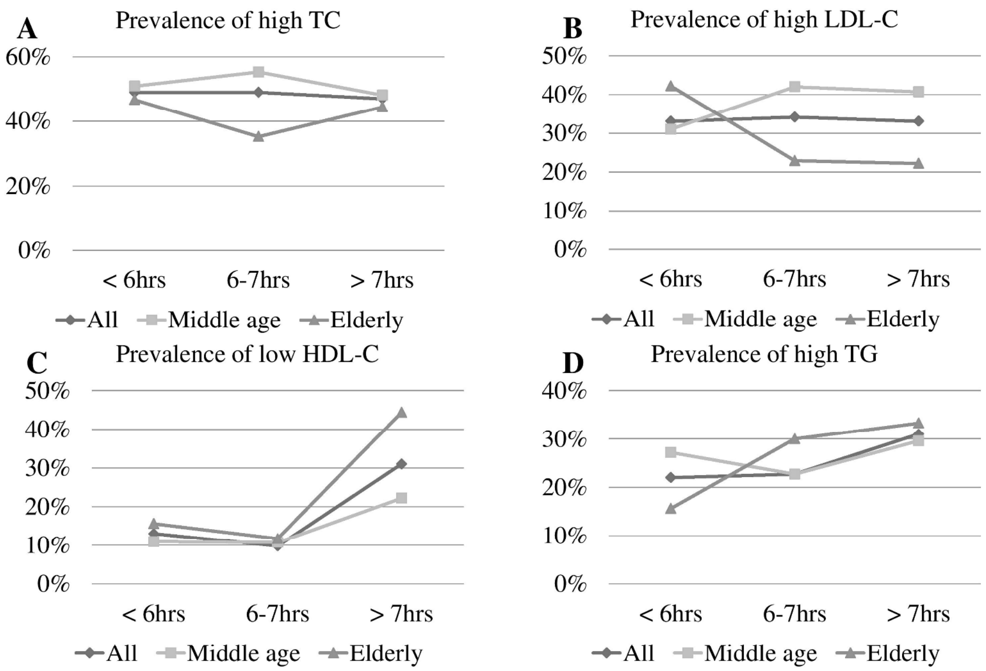


**Figure1.** Prevalence of serum lipid profiles by sleep duration between two age groups (middle age: 50-65 years old; elderly: ≥65 years old)

Figure1. Prevalence of serum lipid profiles by sleep duration between two age groups (middle age: 50-65 years old; elderly: ≥65 years old)

254x190mm (300 x 300 DPI)

**STROBE 2007 (v4) Statement—Checklist of items that should be included in reports of *cross-sectional studies***

| Section/Topic | Item # | Recommendation | Reported on page # |
|---|---|---|---|
| **Title and abstract** | 1 | (*a*) Indicate the study's design with a commonly used term in the title or the abstract | 1-3 |
| | | (*b*) Provide in the abstract an informative and balanced summary of what was done and what was found | 2-3 |
| **Introduction** | | | |
| Background/rationale | 2 | Explain the scientific background and rationale for the investigation being reported | 4-5 |
| Objectives | 3 | State specific objectives, including any prespecified hypotheses | 4-5 |
| **Methods** | | | |
| Study design | 4 | Present key elements of study design early in the paper | 5 |
| Setting | 5 | Describe the setting, locations, and relevant dates, including periods of recruitment, exposure, follow-up, and data collection | 5 |
| Participants | 6 | (*a*) Give the eligibility criteria, and the sources and methods of selection of participants | X |
| Variables | 7 | Clearly define all outcomes, exposures, predictors, potential confounders, and effect modifiers. Give diagnostic criteria, if applicable | 5-7 |
| Data sources/ measurement | 8* | For each variable of interest, give sources of data and details of methods of assessment (measurement). Describe comparability of assessment methods if there is more than one group | 5-6 |
| Bias | 9 | Describe any efforts to address potential sources of bias | *X* |
| Study size | 10 | Explain how the study size was arrived at | 5-6 |
| Quantitative variables | 11 | Explain how quantitative variables were handled in the analyses. If applicable, describe which groupings were chosen and why | 6-7 |
| Statistical methods | 12 | (*a*) Describe all statistical methods, including those used to control for confounding | 7 |
| | | (*b*) Describe any methods used to examine subgroups and interactions | X |
| | | (*c*) Explain how missing data were addressed | X |
| | | (*d*) If applicable, describe analytical methods taking account of sampling strategy | X |

For peer review only

| | | | |
|---|---|---|---|
| | | (*e*) Describe any sensitivity analyses | X |
| **Results** | | | |
| Participants | 13* | (a) Report numbers of individuals at each stage of study—eg numbers potentially eligible, examined for eligibility, confirmed eligible, included in the study, completing follow-up, and analysed | X |
| | | (b) Give reasons for non-participation at each stage | X |
| | | (c) Consider use of a flow diagram | X |
| Descriptive data | 14* | (a) Give characteristics of study participants (eg demographic, clinical, social) and information on exposures and potential confounders | 7-12 |
| | | (b) Indicate number of participants with missing data for each variable of interest | X |
| Outcome data | 15* | Report numbers of outcome events or summary measures | 7-14 |
| Main results | 16 | (*a*) Give unadjusted estimates and, if applicable, confounder-adjusted estimates and their precision (eg, 95% confidence interval). Make clear which confounders were adjusted for and why they were included | 13-14 |
| | | (*b*) Report category boundaries when continuous variables were categorized | X |
| | | (*c*) If relevant, consider translating estimates of relative risk into absolute risk for a meaningful time period | X |
| Other analyses | 17 | Report other analyses done—eg analyses of subgroups and interactions, and sensitivity analyses | X |
| **Discussion** | | | |
| Key results | 18 | Summarise key results with reference to study objectives | 15 |
| Limitations | 19 | Discuss limitations of the study, taking into account sources of potential bias or imprecision. Discuss both direction and magnitude of any potential bias | 17-18 |
| Interpretation | 20 | Give a cautious overall interpretation of results considering objectives, limitations, multiplicity of analyses, results from similar studies, and other relevant evidence | 15-18 |
| Generalisability | 21 | Discuss the generalisability (external validity) of the study results | X |
| **Other information** | | | |
| Funding | 22 | Give the source of funding and the role of the funders for the present study and, if applicable, for the original study on which the present article is based | 19 |

*Give information separately for cases and controls in case-control studies and, if applicable, for exposed and unexposed groups in cohort and cross-sectional studies.

**Note:** An Explanation and Elaboration article discusses each checklist item and gives methodological background and published examples of transparent reporting. The STROBE checklist is best used in conjunction with this article (freely available on the Web sites of PLoS Medicine at http://www.plosmedicine.org/, Annals of Internal Medicine at http://www.annals.org/, and Epidemiology at http://www.epidem.com/). Information on the STROBE Initiative is available at www.strobe-statement.org.

For peer review only
